# An in vitro study of dual drug combinations of anti-viral agents, antibiotics, and/or hydroxychloroquine against the SARS-CoV-2 virus isolated from hospitalized patients in Surabaya, Indonesia

**DOI:** 10.1371/journal.pone.0252302

**Published:** 2021-06-18

**Authors:** Purwati, Andang Miatmoko, Nasronudin, Eryk Hendrianto, Deya Karsari, Aristika Dinaryanti, Nora Ertanti, Igo Syaiful Ihsan, Disca Sandyakala Purnama, Tri Pudy Asmarawati, Erika Marfiani, Yulistiani, Alfian Nur Rosyid, Prastuti Asta Wulaningrum, Herley Windo Setiawan, Imam Siswanto, Ni Nyoman Tri Puspaningsih

**Affiliations:** 1 Stem Cell Research and Development Center, Institute of Tropical Disease, Universitas Airlangga, Mulyorejo, Surabaya, Indonesia; 2 Faculty of Vocations, Universitas Airlangga, Gubeng, Surabaya, Indonesia; 3 Department of Biotechnology, Asia University, Wufeng, Taichung, Taiwan; 4 Faculty of Pharmacy, Universitas Airlangga, Mulyorejo, Surabaya, Indonesia; 5 Rumah Sakit Umum dan Rumah Sakit Khusus Infeksi, Universitas Airlangga, Mulyorejo, Surabaya, Indonesia; 6 Bioinformatic Laboratory, UCoE Research Center for Bio-Molecule Engineering Universitas Airlangga, Surabaya, Indonesia; 7 Department of Chemistry, Faculty of Science and Technology, Universitas Airlangga, Surabaya, Indonesia; Stanford University School of Medicine, UNITED STATES

## Abstract

A potent therapy for the infectious coronavirus disease COVID-19 is urgently required with, at the time of writing, research in this area still ongoing. This study aims to evaluate the in vitro anti-viral activities of combinations of certain commercially available drugs that have recently formed part of COVID-19 therapy. Dual combinatory drugs, namely; Lopinavir-Ritonavir (LOPIRITO)-Clarithromycin (CLA), LOPIRITO-Azithromycin (AZI), LOPIRITO-Doxycycline (DOXY), Hydroxychloroquine (HCQ)-AZI, HCQ-DOXY, Favipiravir (FAVI)-AZI, HCQ-FAVI, and HCQ-LOPIRITO, were prepared. These drugs were mixed at specific ratios and evaluated for their safe use based on the cytotoxicity concentration (CC_50_) values of human umbilical cord mesenchymal stem cells. The anti-viral efficacy of these combinations in relation to Vero cells infected with SARS-CoV-2 virus isolated from a patient in Universitas Airlangga hospital, Surabaya, Indonesia and evaluated for IC_50_ 24, 48, and 72 hours after viral inoculation was subsequently determined. Observation of the viral load in qRT-PCR was undertaken, the results of which indicated the absence of high levels of cytotoxicity in any samples and that dual combinatory drugs produced lower cytotoxicity than single drugs. In addition, these combinations demonstrated considerable effectiveness in reducing the copy number of the virus at 48 and 72 hours, while even at 24 hours, post-drug incubation resulted in low IC_50_ values. Most combination drugs reduced pro-inflammatory markers, i.e. IL-6 and TNF-α, while increasing the anti-inflammatory response of IL-10. According to these results, the descending order of effective dual combinatory drugs is one of LOPIRITO-AZI>LOPIRITO-DOXY>HCQ-AZI>HCQ-FAVI>LOPIRITO-CLA>HCQ-DOX. It can be suggested that dual combinatory drugs, e.g. LOPIRITO-AZI, can potentially be used in the treatment of COVID-19 infectious diseases.

## Introduction

At the end of 2019, a case of pneumonia was diagnosed on the basis of a viral infection in Wuhan, China [1]. The pathogen was identified as a novel enveloped RNA betacoronavirus2, currently referred to as Severe Acute Respiratory Syndrome Coronavirus 2 (SARS-CoV-2), which has a phylogenetic similar to SARS-CoV. Since that time, it has developed into a global pandemic due to Coronavirus SARS-CoV-2, also referred to as COVID-19 [2, 3]. On March 2^nd^ 2020, the Indonesian Ministry of Health reported the first confirmed domestic positive case of SARS-CoV-2. By September 2020, more than 262,000 individuals had been infected with 10,105 cases culminating in death [4].

COVID-19 infection causes severe pneumonia with symptoms such as fever, a persistent cough, and progressive breathing failure associated with respiratory complications. The high hospitalization rate, risk of mortality and lack of a specific established treatment rendered urgent the need for an effective therapy for COVID-19 to be developed. The main viral proteinase has recently been considered positively as a suitable target for drug design against COVID-19 infection due to its vital role in the poly-protein processing necessary for coronavirus reproduction [5].

The term ‘antiviral agents’ refers to the medications prescribed to combat Middle East Respiratory Syndrome (MERS) and SARS pandemics. Interferon α (IFN-α), lopinavir-ritonavir, chloroquine phosphate, ribavirin, and Arbidol have been highlighted in the latest version of the Guidelines for the Prevention, Diagnosis, and Treatment of Novel Coronavirus-induced Pneumonia issued by the Republic of China’s National Health Commission (NHC) as potential treatments for COVID-19 [6]. In addition to antiviral agents, antibiotics such as amoxicillin, azithromycin or fluoroquinolones are also being employed [7] in an attempt to eradicate the SARS-CoV-2 virus. However, given the continuing lack of data regarding their efficacy as a form of COVID-19 therapy, this study aims to evaluate the use of dual combinatory drugs as an antiviral therapy against the SARS-CoV-2 virus, specifically COVID-19, within the Indonesian context.

During the present research, the respective in vitro antiviral activities of Lopinavir-Ritonavir (LOPIRITO), Favipiravir (FAVI), Azithromycin (AZI), Clarithromycin (CLA), Doxycycline (DOXY), and Hydroxychloroquine (HCQ) as dual combinatory drugs at determined ratios were analyzed. These ratios were established based on the plasma concentration of drugs administered at the usual dose during clinical therapy, (see Table 1). However, in many cases, there were limited or even no reports regarding the pharmacokinetic profiles in dual drug combinations.

**Table 1:** Peak plasma concentration of Lopinavir/Ritonavir (LOPIRITO), Azithromycin (AZI), Clarithromycin (CLA), Doxycycline (DOXY), Hydroxychloroquine (HCQ), and Favipiravir (FAVI) after a single oral administration of the drug.

Drugs	Dosage	Peak Plasma Concentration	Reference
Lopinavir/Ritonavir	Oral administration of Aluvia^®^ tablet containing 400/100 mg Lopinavir/Ritonavir twice a day	Lopinavir: 6.9 to 17.7 μg/mL	[8]
Azithromycin	Single oral administration of 500 mg Azithromycin	0.35–0.45 mg/L after	[9]
Clarithromycin	oral administration of 250 and 500 mg Clarithromycin twice a day	1 and 2.41 μg/mL, respectively	[10]
Doxycycline	Single oral administration of 200 mg doxycycline	1.5 to 7.0 μg/ml after oral administration	[11]
Hydroxychloroquine	Single oral administration of 400 mg HCQ sulfate	0.28 to 0.54 μg/mL	[12]
Favipiravir	1600/600 mg twice a day	64.56 μg/mL	[13]

Lopinavir, Ritonavir, and Favipiravir have all been used as antiviral agents which act as virus protease inhibitors [8, 9]. Azithromycin is classified as a macrolide antibiotic which has been used extensively in the treatment of severe respiratory lower tract infections such as pneumonia. It can be employed for preventing secondary infection often resulting from viral infection, thereby avoiding a severe prognosis. Azithromycin has been reported to be an immune modulator and anti-inflammatory agent [10, 11], while also inhibiting virus replication and the cytopathic effect mediated by the Zika virus in Glial cell lines and astrocytes [14]. Moreover, the use of clarithromycin has been regarded in the same manner as that of Azithromycin. Clarithromycin demonstrates a high affinity with the protein target of HIV-1 protease in the molecular docking study which is superior to that of doxycycline due to high hydrophobicity and partition co-efficiency [15]. The combined application of Clarithromycin and antiviral agents, i.e. Oseltamivir or Zanamivir, increased systemic immunity while reducing rates of infection-related relapse in children infected with the influenza virus [16]. Doxycyline, a tetracycline-derived drug, has an inhibitory effect on dengue fever viral replication and reduces the proinflammatory marker IL-6 during viral infections [17]. Consequently, it may prove effective as a form of COVID-19 therapy [18, 19]. Hydroxychloroquine is an aminoquinoline-derivate compound producing fewer severe side effects than chloroquine [20]. It has been employed as an antiviral agent [21, 22] which impedes the viral pre-entry stage, inhibits both viral replication mediated by acidic endocytosis and viral replication through modification of post-translation virus protein, hinders virus maturation via pH modulation, and produces anti-inflammatory effects by reducing IL-6 levels in serum [23].

In this present work, the efficacy of these drugs as a form of COVID-19 therapy was evaluated on Vero cells as viral hosts cultured with SARS-CoV-2 virus isolated from hospitalized patients in Universitas Airlangga Hospital, Surabaya, Indonesia. Furthermore, an analysis of the structure-based computational modelling of ligand-receptor interactions evaluated their potential use as the main protease of SARS-CoV-2 inhibitor [24].

## Material and methods

### Materials

Lopinavir-Ritonavir (LOPIRITO) was produced by Abbott Laboratories (Aluvia®, Chicago, USA); Favipiravir (FAVI) by Toyama Chemical (Fujifilm Group) (Avigan®, Japan); Azithromycin (AZI) tablets by Gentec Pharmaceutical Group (Spain); Clarithromycin (CLA) by Ind Swift Laboratories Limited (India); Doxycycline (DOXY) by Genero Pharmaceuticals (Doxicor®, Indonesia); Hydroxychloroquine (HCQ) by Imedco Djaja (Hyloquin®, Indonesia); and dimethyl sulfoxide by Sigma Aldrich (Singapore). All other reagents and solvents employed in this study were of the highest quality available. Milli-Q water was used in all experiments.

### Virus and cell collection

Vero cells were used for virus inoculation against SARS-CoV-2 isolates in Indonesia. Cells were seeded in a 12-well microplate at a cell density of 5x10^4^ cells/well cultured in Dulbecco’s Modified Eagle’s Medium (DMEM) (Gibco, USA) containing 10% foetal bovine serum (Gibco, USA), 1% penicillin-streptomycin (Gibco, USA) and 1% amphotericin-B (Gibco, USA). Cells were incubated in a CO_2_ incubator at 37°C in a humidified atmosphere of 5% CO_2_ for 24 hours and cultured to reach 80–90% confluence.

SARS-CoV-2 virus isolates were collected from PCR-positive confirmed patients in Universitas Airlangga Hospital, Surabaya. Patient sputum sampling and clinical procedures were performed in accordance with the ethical clearance issued by The Ethics Commission of Universitas Airlangga Hospital (Certificate number 136/KEP/2020 dated April 20, 2020). The sputum of conscious patients was collected in viral transport medium (VTM) containing Gentamycin sulphate (100μg/ml) and Amphotericin B (0.5μg/ml). Further experiments were conducted in the Biosafety Level (BSL)-3 Laboratory at The Institute of Tropical Disease, Universitas Airlangga, Surabaya, Indonesia. In order to isolate the virus, the sputum samples were inserted into a new conical tube, subsequently vortexed for five minutes, and centrifuged at 13,000 rpm for ten minutes. After centrifugation, the supernatant of each sample was extracted for the purposes of further experiments.

### Preparation of drugs solution

Each tablet containing drugs was triturated and mixed until homogenous. Approximately 50 mg equivalent mass of drugs were weighed and added to dimethyl sulfoxide in order to solubilize the drugs. The suspension was sonicated in a water bath for 15 minutes before being added to Rosewell Park Memorial Institute (RPMI) media, sonicated again and vortexed to mix it until homogenous. The suspension was then filtered through a polycarbonate membrane with a pore size of 0.45 μm and then a pore size of 0.22 μm under aseptic conditions. The filtrate was mixed with 10% foetal bovine serum and penicillin streptomycin before being vortexed to produce a homogenous mixture to be used as a stock solution. The samples were prepared by diluting the stock solution of each drug with RPMI complete media at an appropriate level of dilution to produce a determined concentration. The dual combinatory drugs mixtures were prepared by mixing appropriate amounts of two drug stock solutions in order to produce a final concentration at the required level. The combinatory drugs were evaluated at both constant and non-constant ratios to evaluate their effects on the cytotoxicity, including; antagonistic, synergistic, or additive. A constant ratio of the mixture was achieved by adding drug solutions at the same ratio, thereby increasing each drug concentration, to produce dose escalation. In contrast, at a non-constant ratio, a fixed determined concentration of drug was added to increased doses of other drug solution in order to produce different levels of drug concentration.

### Cytotoxicity assay for dual combinatory drugs

The cytotoxic concentration (CC_50_) of drugs was performed by means of MTT assay at the Stem Cell Research and Development Center, Universitas Airlangga using human umbilical cord mesenchymal stem cells which had been obtained from human placenta tissue as approved by the Ethical Committee of Universitas Airlangga Hospital (Certificate number 101/KEH/2019 dated January 10, 2019). The cells were prepared as the primary cell culture and used for the cytotoxicity assay because of their sensitivity to chemicals. Cells were seeded into 96-well microplates at a concentration of 1x10^3^ cells/well in 100 μL Alpha Minimum Essentials Medium (α-MEM, Gibco, USA) supplemented with 10% foetal bovine serum, 1% penicillin-streptomycin and 1% amphotericin-B. The plates were then incubated in a CO_2_ incubator at 37°C with 5% CO_2_ for 24 hours, at which point, the supernatant was replaced with α-MEM containing drugs at each concentration and incubated for a further 48 hours. Approximately 25μL of 3-(4,5-Dimethylthiazol-2-yl)-2,5-diphenyl tetrazolium-bromide (MTT) reagent at a concentration of 5mg/mL was subsequently added to each well and incubated for four hours at 37°C with 5% CO_2_. Purple formazan crystals were formed and observed under an inverted microscope. Dimethyl sulfoxide was added to each well with the complete solubilisation of formazan crystals subsequently being observed. The greater the number of formazan crystals formed, the lower the toxicity of the samples which were read for optical density of formazan using a multi reader at a measurement wavelength of 595 nm (Promega Glomax, USA). The CC_50_ value was analyzed by CompuSyn software (the ComboSyn Inc., accessed from www.combosyn.com).

### Virus inoculation and antiviral assay for dual combinatory drugs

Vero cells obtained from Elabscience® (Catalog No. EP-CL-0242, USA) were seeded in a 12-well plate and confirmed as reaching 80–90% confluence on the day of virus inoculation. The culture medium was removed and the cells were then added to RPMI media containing SARS-CoV-2 isolates, previously diluted with RPMI media at a ratio of 1:2. In this study, about 2,000 virus copies were added to 50,000 cells of Vero cells, with a multiplicity of infection (MoI) degree of 0.04. The plate was gently agitated for 30 minutes and incubated at 37°C, 5% CO_2_ for 24 hours. About 3 mL of complete culture medium were subsequently added to the plate and incubated at 5% CO_2_ 37°C for 24 hours, at which point 3 mL of RPMI media containing a drug combination were introduced and incubated at 5% CO_2_ 37°C for 24, 48, and 72 hours. The drug mixtures were prepared at appropriate weight constant ratios selected on the basis of the optimum safety profiles in the cytotoxicity study. The Vero cells were observed post-treatment to observe the cytopathic effects, including; the rounding and detachment of cells. Moreover, the IC_50_ values were determined in order to quantify antiviral activity by measuring the proviral load in each well. The determination of the proviral load was performed by means of a Seegene COVID-19 detection Kit (Beijing, China) which detected three target genes, i.e. N-gene, E-gene and RdRP-gene. Amplification and data acquisition were carried out using the ABI Prism 7500 Sequence detector system (Applied Biosystems, USA). The IC_50_ value was further analyzed using CompuSyn software (The ComboSyn Inc., accessed from www.combosyn.com).

### Measurement of IL-6, IL-10 and TNF-α levels of virus-infected Vero cells incubated with dual combinatory drugs

To enable measurement of IL-6, IL-10 and TNF-α levels, the culture medium of the treated cells was collected in sterile micro-tubes and centrifuged at 3,500 rpm for 20 minutes. The supernatants were carefully collected and diluted with aquadest at a 1:5 volume ratio and vortexed until homogenous. The samples were deposited onto a well-plate, added to ELISA reagents (Bioassay Technology Laboratory, Shanghai, China), and incubated at 37°C for 60 minutes. Reagent substrate solution was then added to the well and incubated for ten minutes at 37°C. The samples were measured for antigen concentration using the optical density (OD) plotted into the standard curves of IL-6, IL-10, and TNF-α.

### Molecular docking study of drugs against main protease of SARS-CoV-2 virus

The molecular docking study was carried out by using Schrodinger Maestro 2019–2 Maestro software including protein preparation, ligand preparation, grid generation and receptor-ligand docking. The Linux operating system was used for the computational study. Ligands (Lopinavir, Ritonavir, Favipiravir, Azithromycin, Clarithromycin, Doxycycline, and Hydroxychloroquine) were downloaded from the NCBI (http://www.ncbi.nlm.nih.gov/pccompound). The crystal Structure of SARS-CoV-2 main protease, PDB ID: ALU6 was retrieved from the Protein Data Bank (PDB) (https://www.rcsb.org/).

The main protease protein was prepared for a docking study by using in Schrodinger 2019–2 Maestro software. All ligand compounds were prepared using LigPrep, which can produce low energy isomer of the ligand in optimization by using the OPLS_2005 force field. The OPLS_2005 force field was used for generating Grid on protein receptors. Schrodinger 2019–2 version was used to predict the binding affinity, ligand competence, and inhibitory candidate to the protein by performing rigid, flexible docking. The ligands were docked with generated Grid of receptor protein PDB ID: ALU6 The optimal ligand selection for the receptor was done based on the docking score.

#### Preparation of ligands and receptors

**Fig 1:**
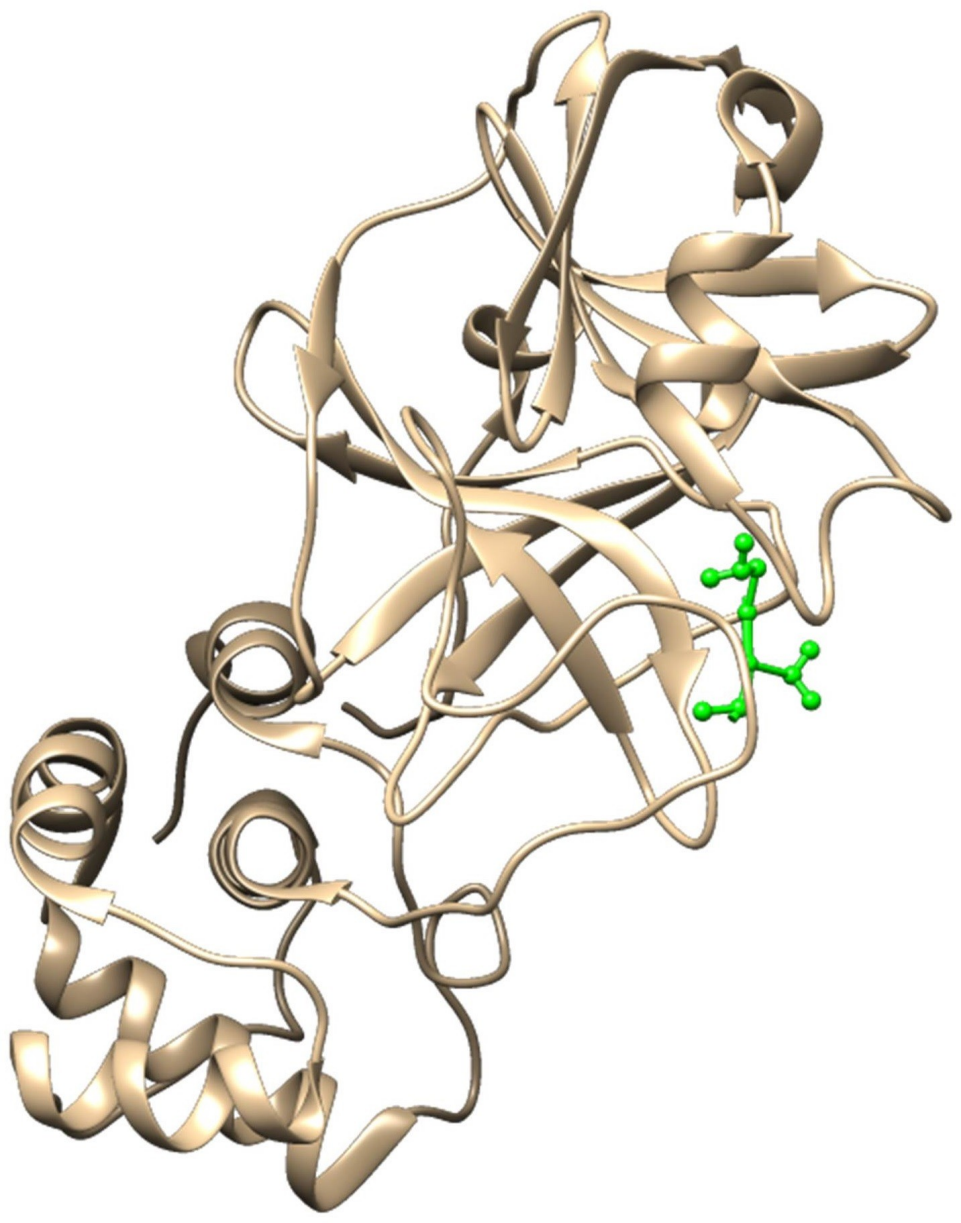
Image representation of ligand-receptor complex.

*Ligand-receptor complex*. The complex in the form of a crystal structure consisting of native ligands and receptors was downloaded from the Protein Data Bank (PDB) server at the web address https://www.rcsb.org with ID 6LU7 [25]. 6LU7 protein structure consists of two chains (A and C). The Main protease (Mpro) is in the A chain (shown in brown), while the native ligand appears as blue in the C chain, as presented in Fig 1.

The receptors and ligands from the resulting crystalline structure did not undergo geometric optimization treatment because they were obtained from the actual structure. For the purposes of the docking procedure, the ligands of this crystal were given a partial charge of the atom using the Austin Model 1 semi-empirical method with Bond Charge Correction (AM1-BCC) [26], while the receptor partial charge was calculated by means of a molecular mechanics approach with a force field of ff14SB [27].

*Preparation of candidates as ligands*. A sketch of the molecular structure of the ligand was produced using the ChemDraw Professional version 17 program. This structural sketch was still 2-dimensional with the result that a 3-dimensional structure had to be made. This structure was formed by calculations using the MM + molecular mechanics method to quickly obtain a 3-dimensional structure. The calculations were performed using a HyperChem 6 program. The structure of the calculation using the molecular mechanics method was then refined using a semi-empirical Parametric Model number 3 (PM3) quantum mechanics calculation. The calculations were completed using Gaussian 16 software. The partial atomic charge of each ligand was calculated through application of the AM1-BCC semi-empirical method.

#### Construction surface and receptor spheres

The receptor surface (molecular surface, ms) consisting of a number of cluster spheres was created and calculated using the dms module which is part of the Dock 6 program [28]. The active side of the M^pro^ was determined based on the native ligand position in the cluster. This active side location was used as the basis for the construction of the simulation box. The degree of margin for the formation of the simulation box was 10 Å.

#### Creating a simulation box

**Fig 2:**
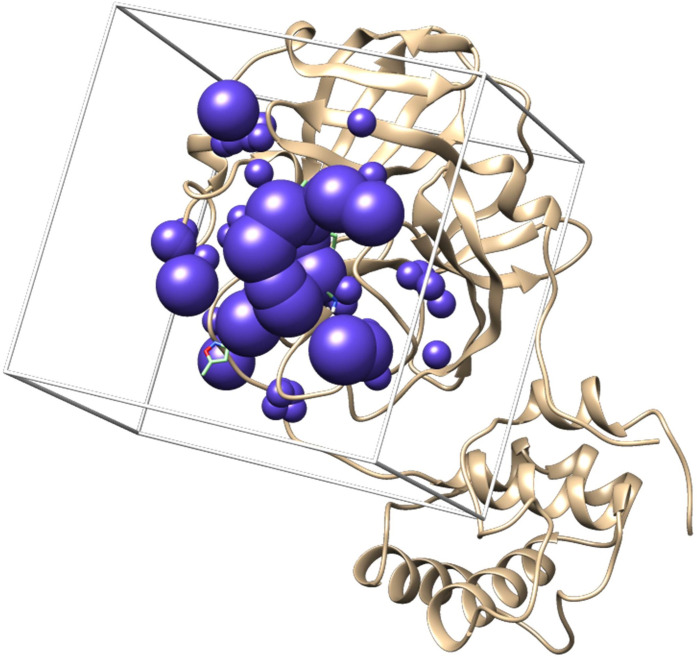
The position of the simulation box, native ligand, and cluster of spheres relative to the receptor.

Depending on the position of the native ligand, a simulation box was built around it in the shape of a cube. The position of the simulation box, native ligand, and cluster of spheres relative to the receptor can be seen in the Fig 2.

#### Validation of docking parameters

The parameters to be employed in docking the candidate to the receptor were validated by redocking the native ligand to the receptor. An effective docking parameter must be able to return the native ligand to its original position with a maximum root mean square deviation (rmsd] tolerance of 2 Å [26]. The docking parameter validation resulted in an rmsd of 1.725 Å, indicating that use of the docking parameters at the docking stage for candidate ligands was feasible.

## Results

### Characterization of human umbilical cord mesenchymal stem cells

**Fig 3:**
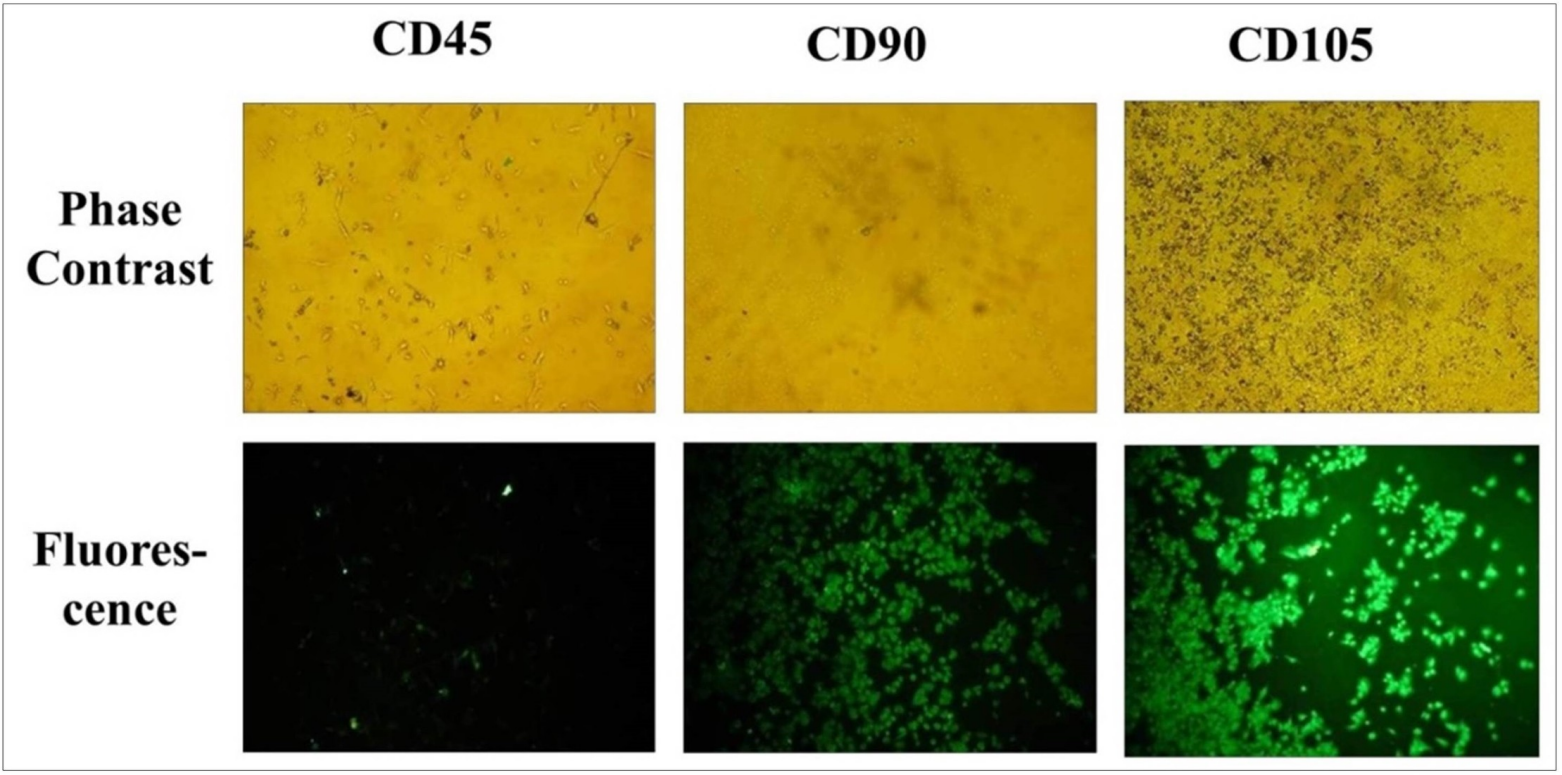
Phase contrast and fluorescence images of human umbilical cord stem cells stained with anti-CD45, CD90, and CD105 antibody and CF555-labelled secondary antibody observed under a fluorescence microscope at a magnification of 100x.

For the cytotoxicity assay of combinatory drugs, the primary cell cultures of human umbilical cord mesenchymal stem cells were used as the experimental cells. From the contents of Fig 3, it is clear that, as previously reported [28–31], the stem cells were well differentiated as indicated by immunocytochemistry assays conducted using CD45, CD90, and CD105 antibodies.

### Cytotoxicities of dual drug combination of LOPIRITO-AZI in mesenchymal stem cells

In this study, the cytotoxicity assay was evaluated for single and dual combinatory drugs during a period of 48 hours of drug incubation. This assay was intended to evaluate the toxicity of dual combinatory drugs on normal cells. The combination ratios were calculated taking into consideration the usual therapeutic doses and plasma peak concentrations of the drugs. To determine this cytotoxicity, the drugs were mixed at both constant and non-constant ratios.

**Fig 4:**
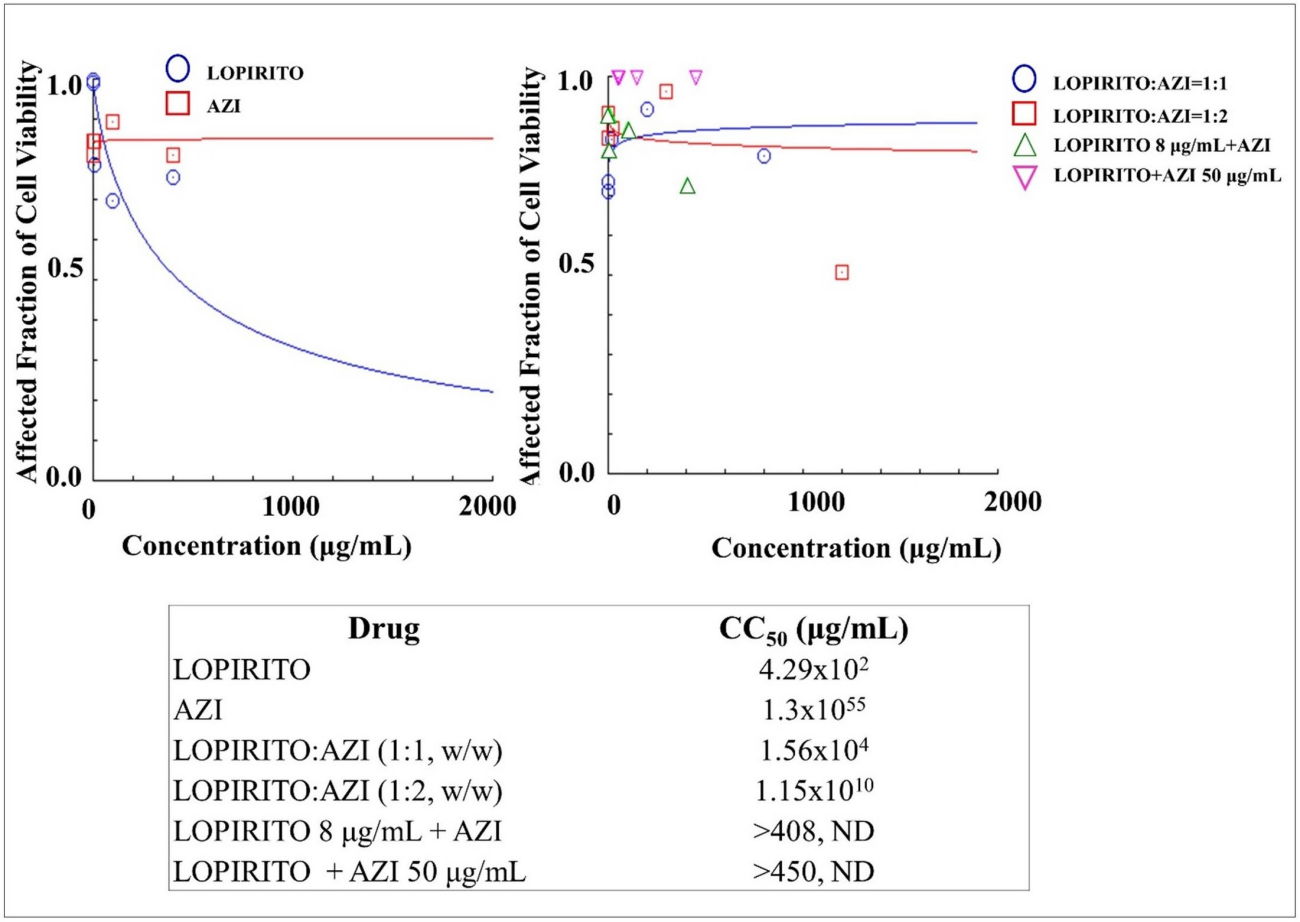
**The cytotoxicity of Lopinavir-Ritonavir (LOPIRITO) and Azithromycin (AZI) as a single drug (left) and dual drug combination at constant and non-constant ratios (right) analysed by CompuSyn Software (n = 3).** At non-constant ratios of LOPIRITO 8 μg/mL + AZI, LOPIRITO was added at a concentration of 8 μg/mL to each increased level of AZI, i.e. 0.2, 2, 10, 100, and 400 μg/mL. On the other hand, AZI was then added at a concentration of 50 μg/mL to each increased level of LOPIRITO, i.e. 0.2, 2, 10, 100, and 400 μg/mL to produce LOPIRITO + AZI 50 μg/mL.

The evaluation of LOPIRITO and AZI in the stem cells showed that AZI had relatively non-toxic properties compared to those of LOPIRITO, while the CC_50_ values were 1.3x10^55^ μg/mL for AZI and 4.29x10^2^ μg/mL for LOPIRITO, as shown in Fig 4. The combination of LOPIRITO and AZI at constant weight ratios of 1:1 and 1:2 respectively, and non-constant ratios resulted in decreases in the degree of cytotoxicity. These were much safer than LOPIRITO as indicated by their higher CC_50_ values. These results indicate that a combination of both drugs negates the side effects of each single one, possibly producing an antagonist effect.’

### Cytotoxicities of dual drug combination of LOPIRITO-CLA in mesenchymal stem cells

**Fig 5:**
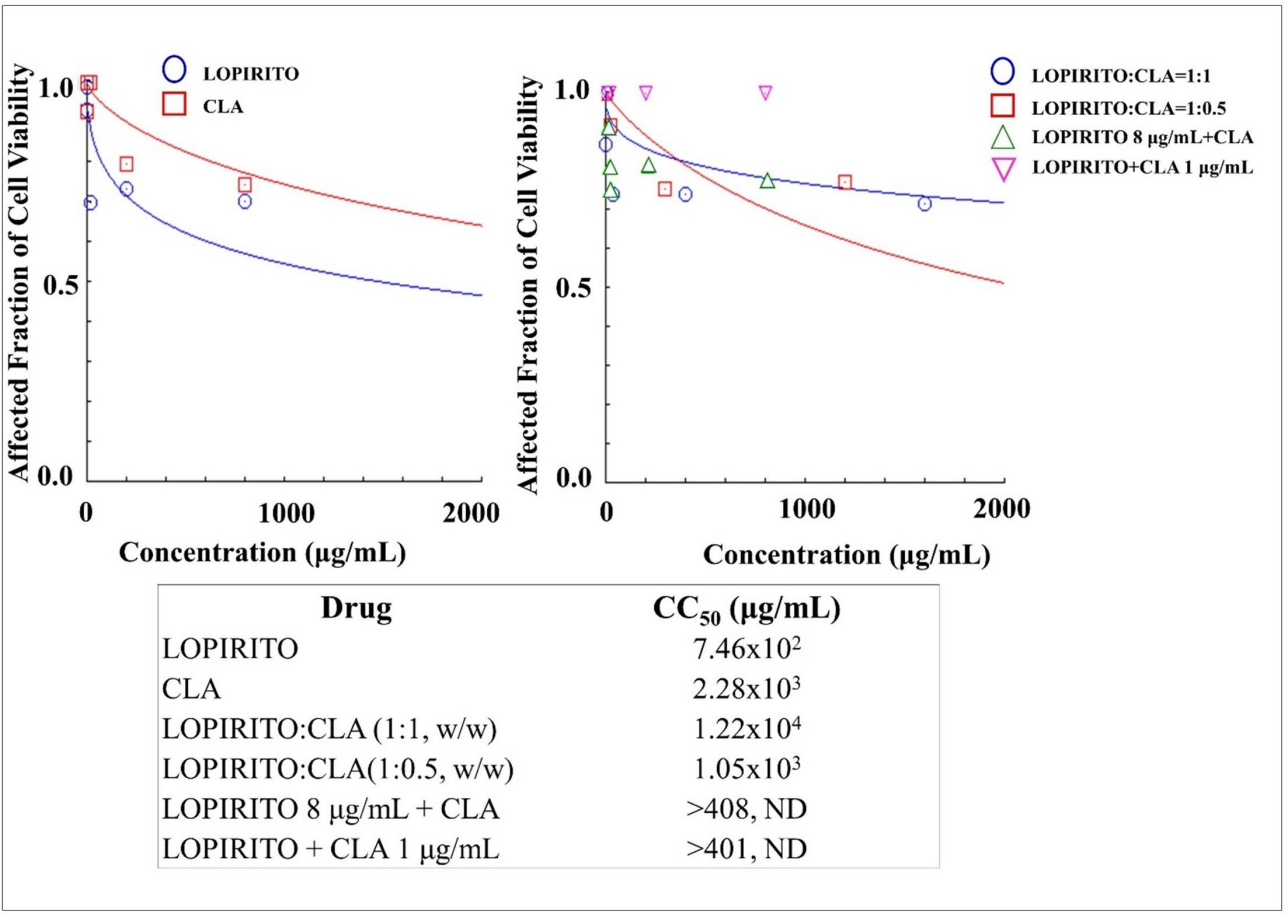
**The cytotoxicities of Lopinavir-Ritonavir (LOPIRITO) and Clarithromycin (CLA) as a single drug (left) and dual drug combination in constant and non-constant ratios (right) analysed by using CompuSyn Software (n = 3).** At non-constant ratios of LOPIRITO 8 μg/mL + CLA, LOPIRITO was added at a concentration of 8 μg/mL to each increased levels of CLA i.e. 0.2, 2, 10, 100, and 400 μg/mL. On the other hand, CLA was then added at a concentration of 1 μg/mL to each increased levels of LOPIRITO i.e. 0.2, 2, 10, 100, and 400 μg/mL to produce LOPIRITO + CLA 1 μg/mL.

The results of a cytotoxicity assay indicated that LOPIRITO was relatively more toxic to the cells than CLA as indicated by their CC_50_ values as a single drug which were 7.46x10^2^ μg/mL and 2.28x10^3^ μg/mL respectively, as shown in Fig 5. Moreover, the dual drug combination of LOPIRITO:CLA at the weight ratio of 1:1 had a high CC_50_ value of 1.22x10^4^ μg/mL, indicating that this combination reduced the toxicity of both drugs in the stem cells.

### Cytotoxicities of dual drug combination of LOPIRITO-DOXY in mesenchymal stem cells

**Fig 6:**
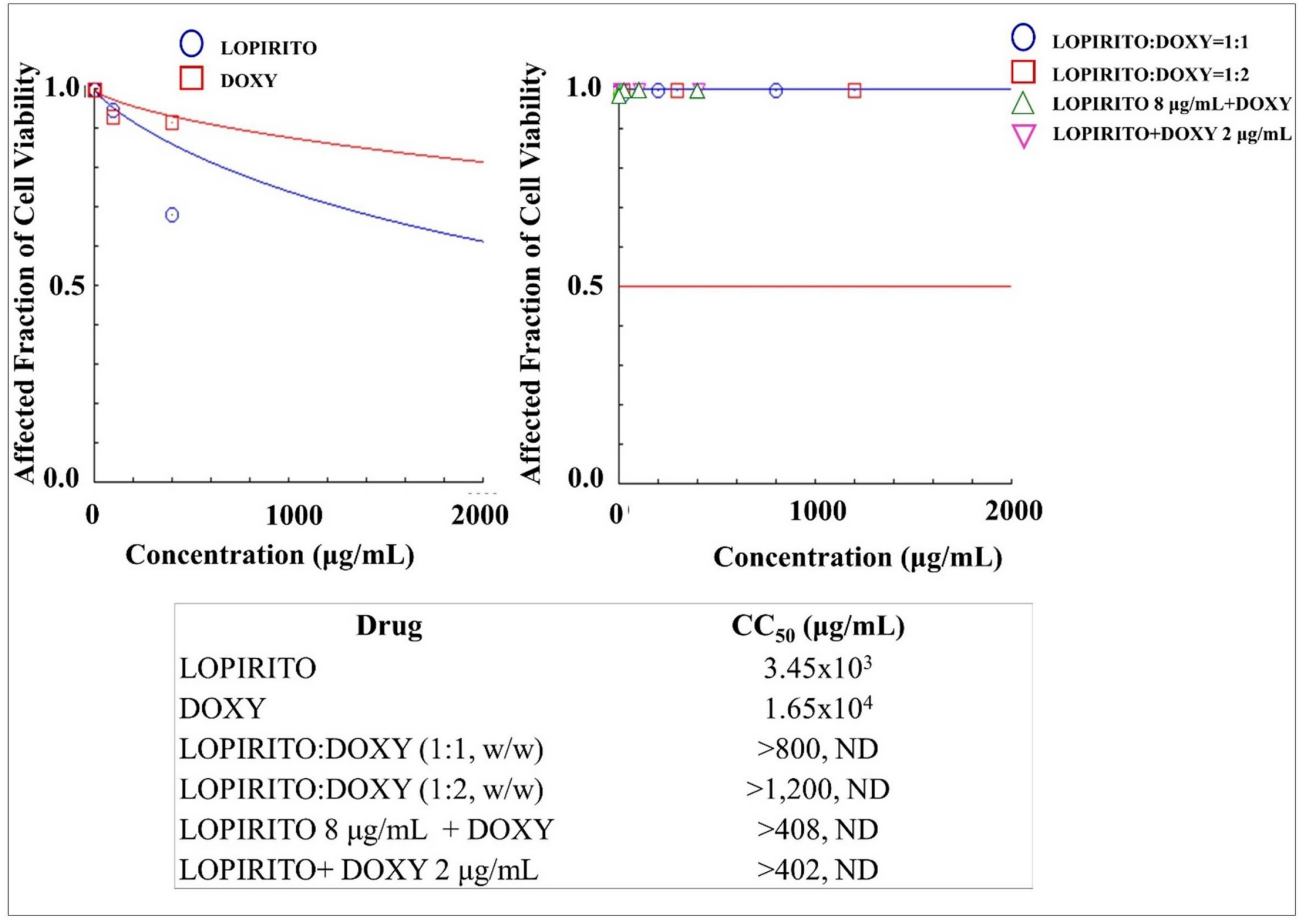
**The cytotoxicities of Lopinavir-Ritonavir (LOPIRITO) and Doxycycline (DOXY) as a single drug (left) and dual drug combination in constant and non-constant ratios (right) analysed by using CompuSyn Software (n = 3).** At non-constant ratios of LOPIRITO 8 μg/mL + DOXY, LOPIRITO was added at a concentration of 8 μg/mL to each increased levels of DOXY i.e. 0.2, 2, 10, 100, and 400 μg/mL. On the other hand, DOXY was then added at a concentration of 2 μg/mL to each increased levels of LOPIRITO i.e. 0.2, 2, 10, 100, and 400 μg/mL to produce LOPIRITO + DOXY 2 μg/mL.

Further evaluation was conducted for the dual combination of LOPIRITO and DOXY. The results showed that LOPIRITO has higher cytotoxicity than DOXY, as shown in Fig 6. The dual combination of LOPIRITO and DOXY, at both constant and non-constant ratios, resulted in significantly higher CC_50_ values (until undetected) than those of single drugs which were 3.45x10^3^ μg/mL and 1.65x10^4^ μg/mL respectively for LOPIRITO and DOXY. This indicated that these combinations reduced drug toxicity in the stem cells.

### Cytotoxicities of dual drug combination of HCQ-AZI in mesenchymal stem cells

**Fig 7:**
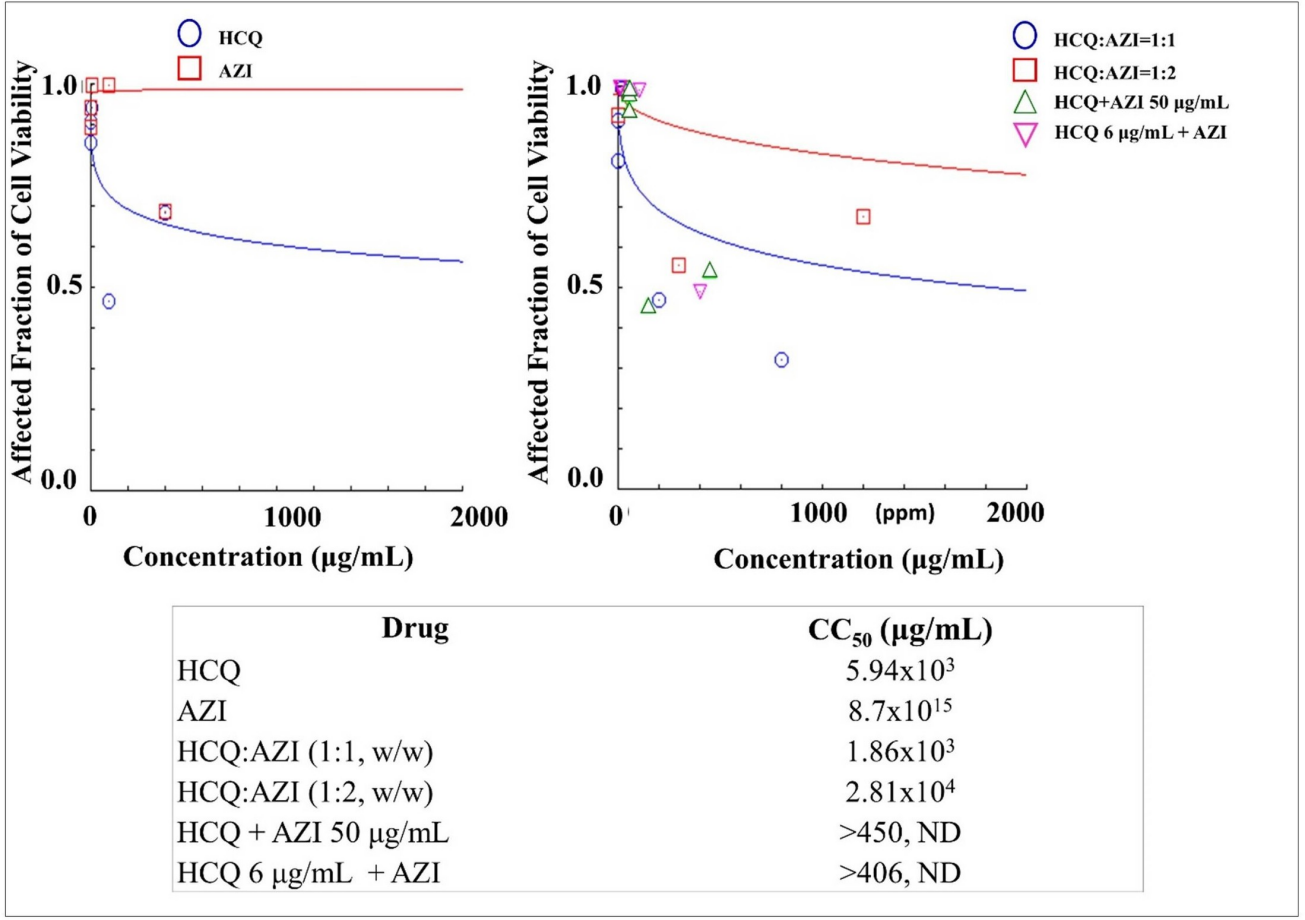
**The cytotoxicities of Hydroxychloroquine (HCQ) and Azithromycin (AZI) as a single drug (left) and dual drug combination in constant and non-constant ratios (right) analysed by using CompuSyn Software (n = 3).** At non-constant ratios of HCQ + AZI 50 μg/mL, AZI was added at a concentration of 50 μg/mL to each increased levels of HCQ i.e. 0.2, 2, 10, 100, and 400 μg/mL. On the other hand, HCQ was then added at a concentration of 6 μg/mL to each increased levels of AZI i.e. 0.2, 2, 10, 100, and 400 μg/mL to produce HCQ 6 μg/mL + AZI.

The cytotoxicity assay was also evaluated for dual combination of HCQ and AZI. As shown in Fig 7, HCQ produced higher cytotoxicity than AZI. Combining these drugs increased the CC_50_ values resulting in a lower toxic effect than that of HCQ. The dual combination drug at a ratio of 1:2 for HCQ and AZI produced the lowest cytotoxicity in the stem cells in which the CC_50_ was 2.81x10^4^ μg/mL, thus providing for its potential use in an anti-viral study of COVID-19.

### Cytotoxicities of dual drug combination of HCQ-DOXY in mesenchymal stem cells

**Fig 8:**
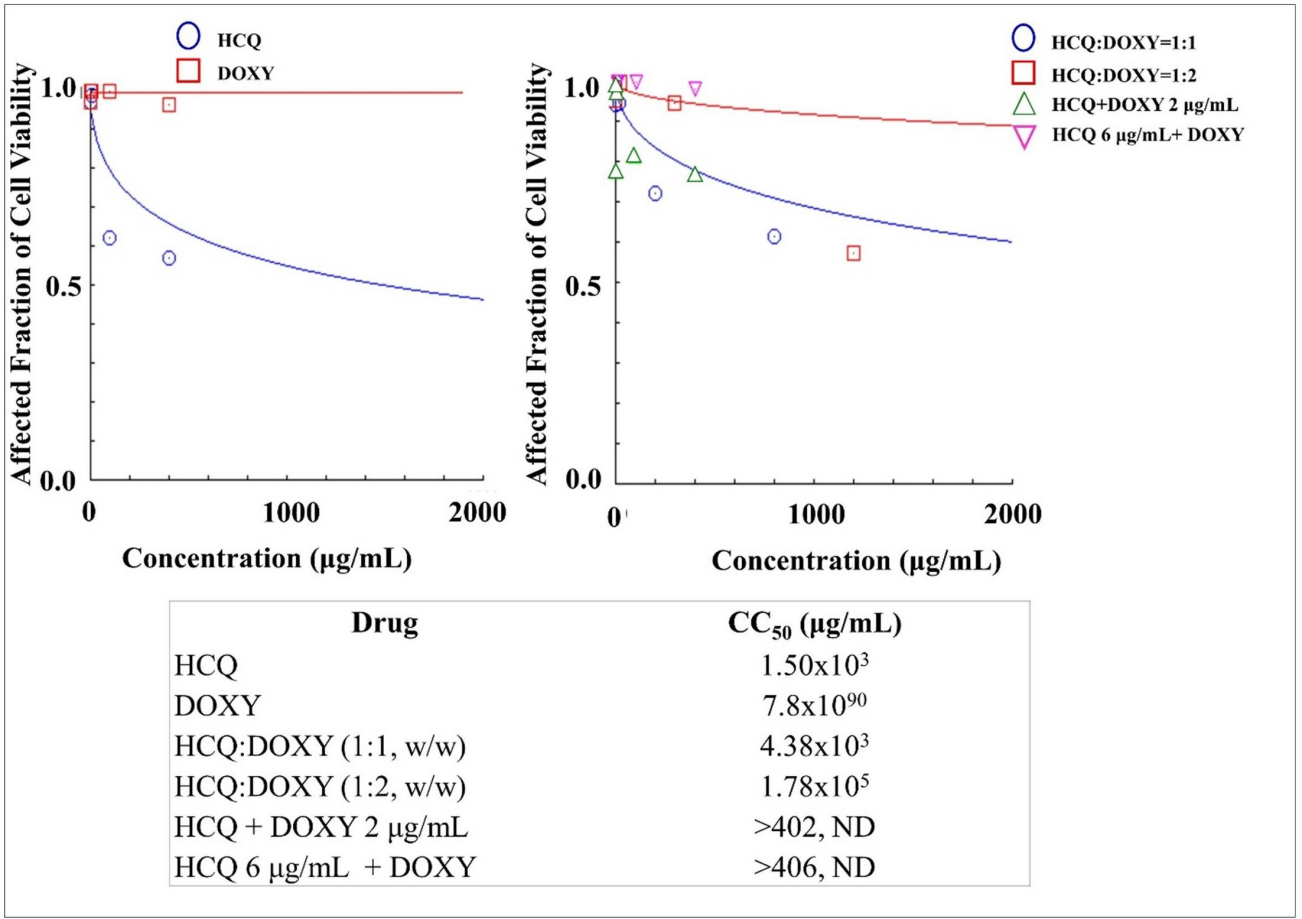
**The cytotoxicities of Hydroxychloroquine (HCQ) and Doxycycline (DOXY) as a single drug (left) and dual drug combination in constant and non-constant ratios (right) analysed by using CompuSyn Software (n = 3).** At non-constant ratios of HCQ + DOXY 2 μg/mL, DOXY was added at a concentration of 2 μg/mL to each increased levels of HCQ i.e. 0.2, 2, 10, 100, and 400 μg/mL. On the other hand, HCQ was then added at a concentration of 6 μg/mL to each increased levels of DOXY i.e. 0.2, 2, 10, 100, and 400 μg/mL to produce HCQ 6 μg/mL + DOXY.

The use of HCQ was combined with DOXY to evaluate its safety when used during antiviral studies. As can be seen in Fig 8, HCQ had higher cytotoxicity than DOXY. Furthermore, the results showed that the dual drug combination produced lower toxicity in the stem cells than that of a single HCQ-based treatment. The CC_50_ values of a combination of HCQ-DOXY at respective weight ratios of 1:1 and 1:2 were 4.37x10^3^ μg/mL and 1.77x10^5^ μg/mL, while the HCQ was 1.50x10^3^ μg/mL.

### Cytotoxicities of dual drug combination of FAVI-AZI in mesenchymal stem cells

**Fig 9:**
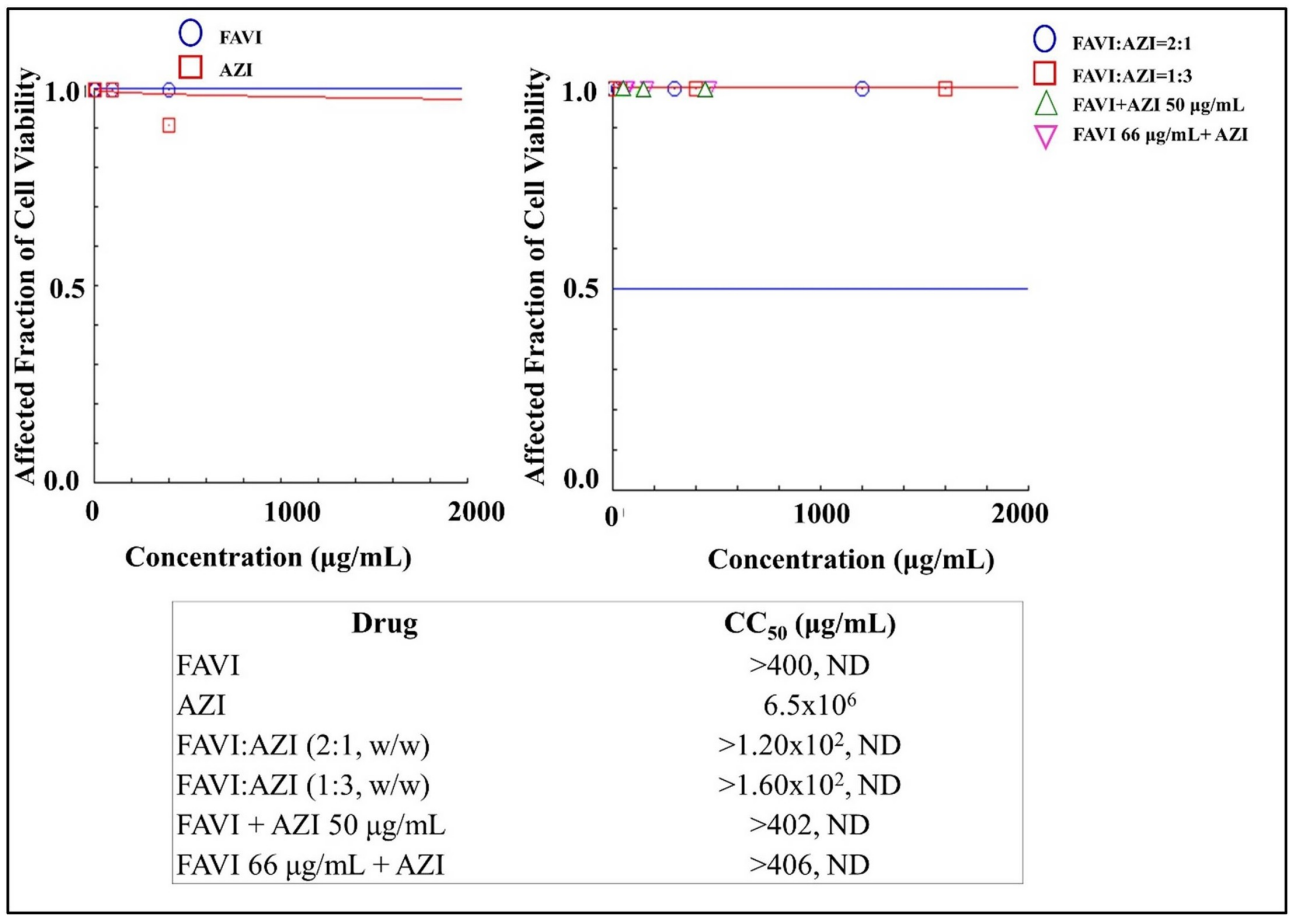
**The cytotoxicities of Favipiravir (FAVI) and Azithromycin (AZI) as a single drug (left) and dual drug combination in constant and non-constant ratios (right) analysed by using CompuSyn Software (n = 3).** At non-constant ratios of FAVI + AZI 50 μg/mL, AZI was added at a concentration of 50 μg/mL to each increased levels of FAVI i.e. 0.2, 2, 10, 100, and 400 μg/mL. On the other hand, FAVI was then added at a concentration of 66 μg/mL to each increased levels of AZI i.e. 0.2, 2, 10, 100, and 400 μg/mL to produce FAVI 66 μg/mL + AZI.

The use of FAVI and AZI in an antiviral study of COVID-19 was initially evaluated for cytotoxicity against primary cultured stem cells. As shown in Fig 9, the results indicated that both FAVI and AZI, administered either as a single drug or in dual combination, produced very low cytotoxicity effects. It could be confirmed that FAVI and AZI were considered drugs not harmful to mesenchymal stem cells.

### Cytotoxicities of dual drug combination of HCQ-FAVI in mesenchymal stem cells

**Fig 10:**
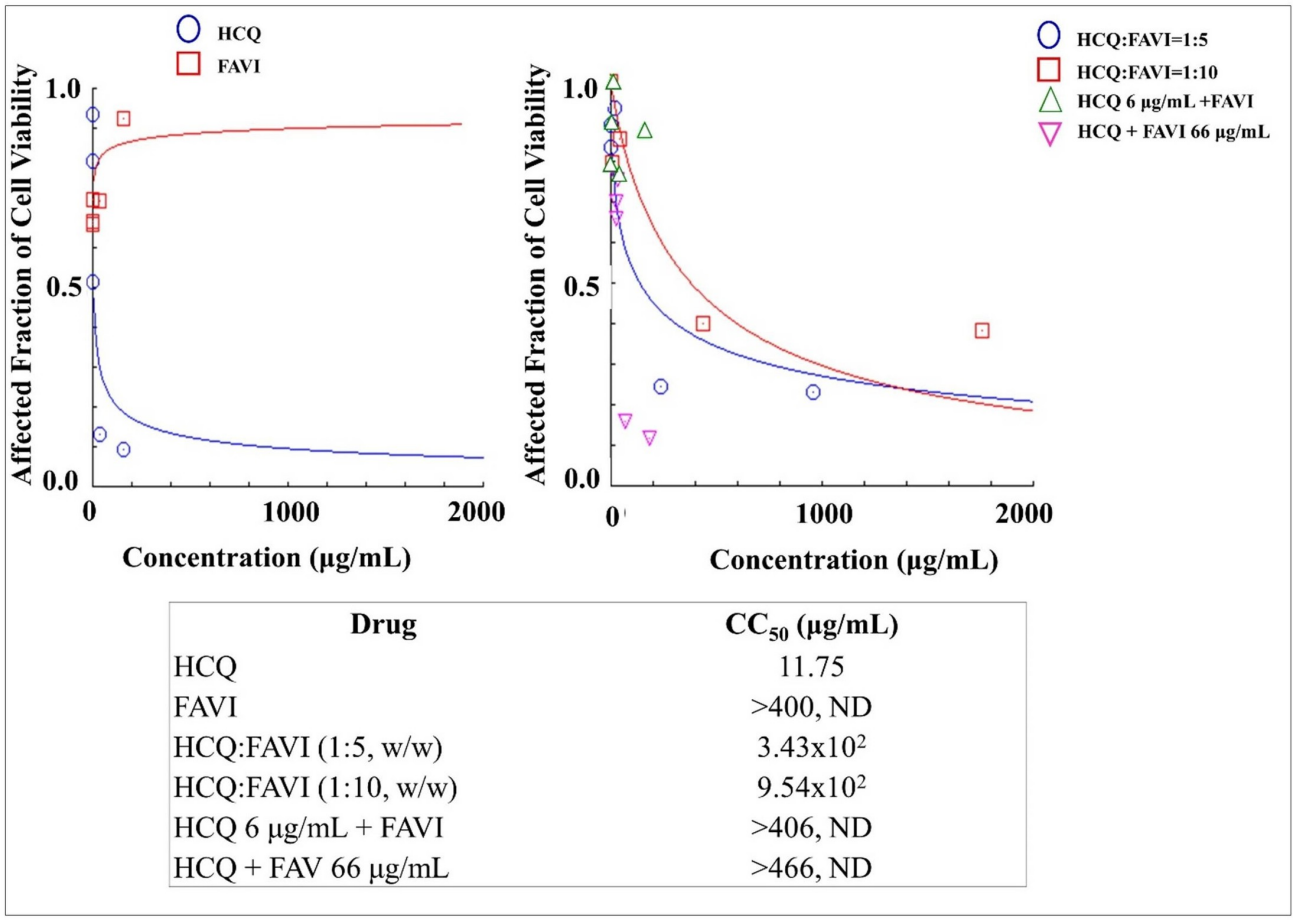
**The cytotoxicities of Hydroxychloroquine (HCQ) and Favipiravir (FAVI) as a single drug (left) and dual drug combination in constant and non-constant ratios (right) analysed by using CompuSyn Software (n = 3).** At non-constant ratios of HCQ 6 μg/mL + FAVI, HCQ was added at a concentration of 66 μg/mL to each increased levels of FAVI i.e. 0.2, 2, 10, 100, and 400 μg/mL. On the other hand, FAVI was then added at a concentration of 66 μg/mL to each increased levels of HCQ i.e. 0.2, 2, 10, 100, and 400 μg/mL to produce HCQ + FAVI 66 μg/mL.

The HCQ was also evaluated for its combination with FAVI. As presented in Fig 10, as a single drug, HCQ produced more intense cytotoxic effects in the mesenchymal stem cells than did FAVI whose CC_50_ value of HCQ was 11.75 μg/mL. Combining HCQ with FAVI reduced the toxicity resulting in higher CC_50_ values of the HCQ-FAVI combination which were 343 μg/mL and 954 μg/mL for HCQ-FAV mixed at the ratios of 1:5 and 1:10 respectively.

### Cytotoxicities of dual drug combination of HCQ-LOPIRITO in mesenchymal stem cells

**Fig 11:**
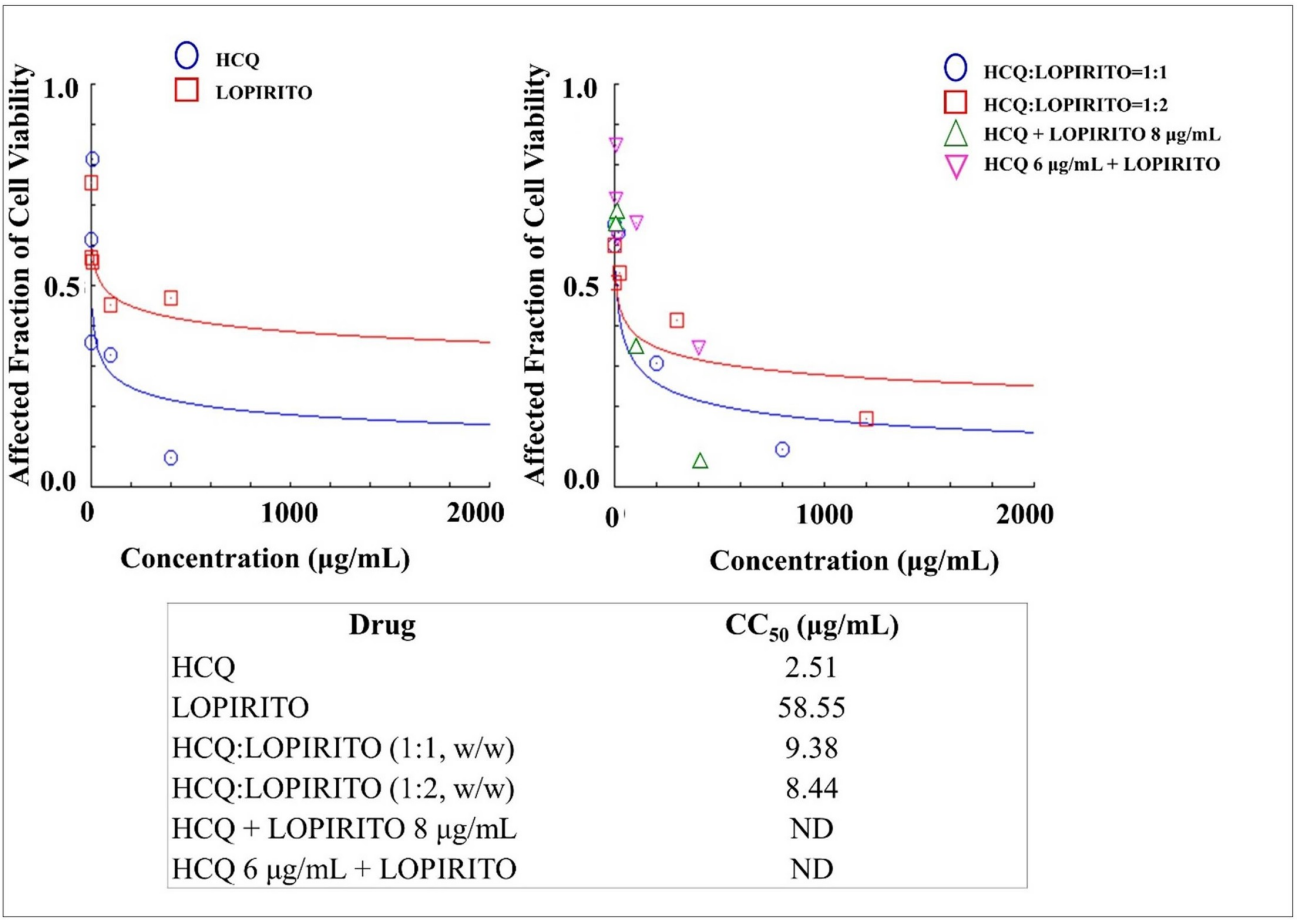
**The cytotoxicities of Hydroxychloroquine (HCQ) and Lopinavir-Ritonavir (LOPIRITO) as a single drug (left) and dual drug combination in constant and non-constant ratios (right) analysed by using CompuSyn Software (n = 3).** At non-constant ratios of HCQ + LOPIRITO 8 μg/mL, LOPIRITO was added at a concentration of 8 μg/mL to each increased levels of HCQ i.e. 0.2, 2, 10, 100, and 400 μg/mL. On the other hand, HCQ was then added at a concentration of 6 μg/mL to each increased levels of LOPIRITO i.e. 0.2, 2, 10, 100, and 400 μg/mL to produce HCQ 6 μg/mL + LOPIRITO.

HCQ was dually combined with LOPIRITO and evaluated for its safe use against mesenchynal stem cells. In this assay, HCQ and LOPIRITO produced relatively low CC_50_ values of 2.51 and 58.55 μg/mL and were considered potentially toxic drugs and combinations as shown in Fig 11. The dual combination of HCQ and LOPIRITO produced higher CC_50_ values than single HCQ, i.e. 9.38 μg/mL and 8.45 μg/mL, for HCQ:LOPIRITO combined at weight ratios of 1:1 and 1:2. respectively. However, they were still more toxic than LOPIRITO.

### Antiviral activity in Vero cells infected with SARS-CoV-2-isolated human virus

**Fig 12:**
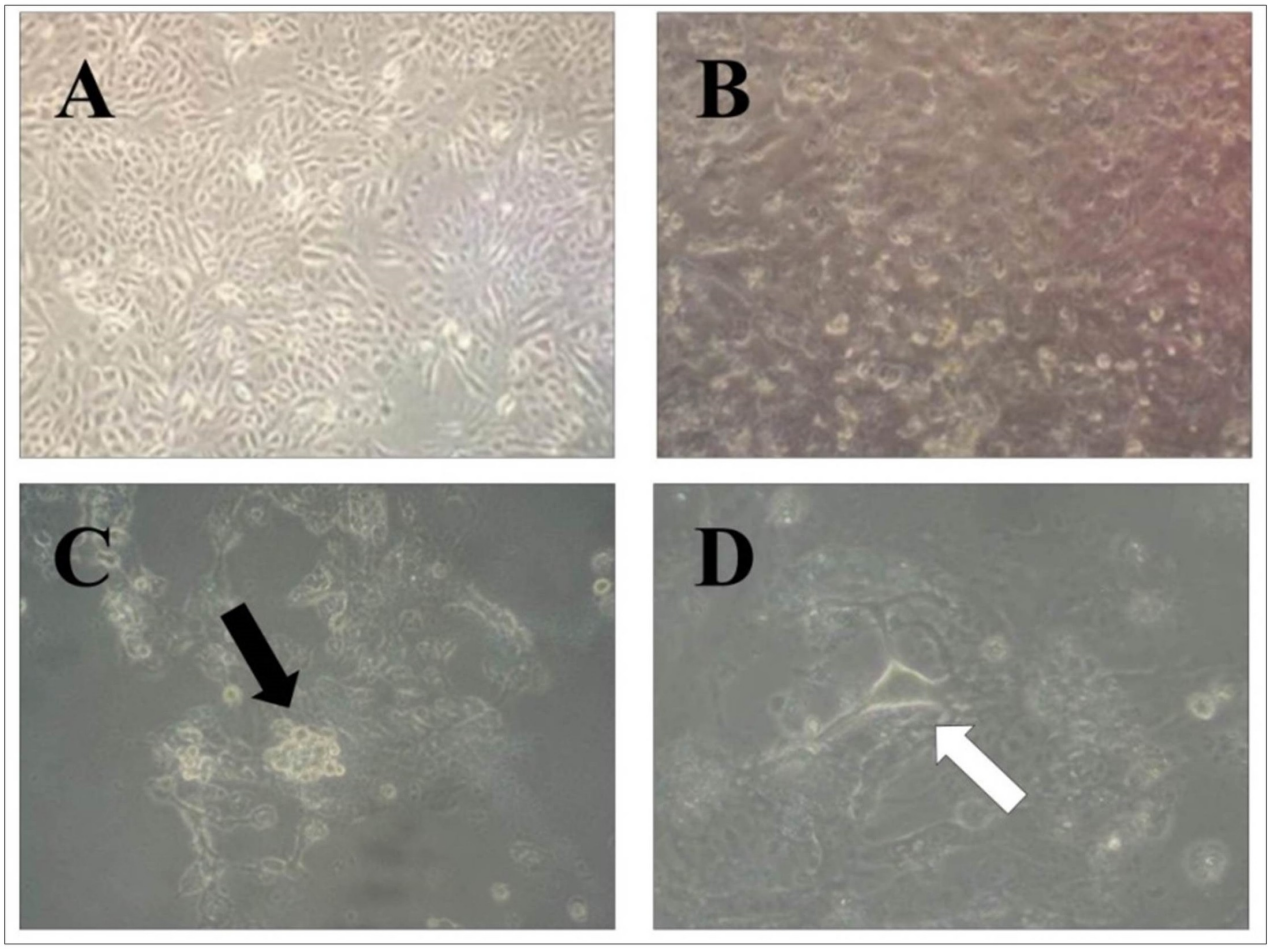
The photomicrographs of morphology changes of Vero cells before virus inoculation (A), at 24-h (B), 48-h (C), and 72-h (D) after virus inoculation observed at a magnification of 100x. The black arrow shows a giant cell formation and the white arrow indicates a cytopathic effect.

After cytotoxic evaluation of dual drug combination in mesenchymal stem cells, the drugs were subsequently assessed for antiviral activities against the SARS-CoV-2 virus isolated from patients in Universitas Airlangga Hospital. The Vero cells were inoculated with the virus which led to certain changes in their morphology indicating that the virus had successfully infected them. Fig 12 contains the typical formations of virus-infected cells observed at 24, 48, and 72 hours post-inoculation. At 24 hours post-inoculation, the presence of groups or colonies of detached cells indicated that they were dead. Furthermore, the formation of giant cells was observed in the 48 hours followed by a cytopathic effect clearly evident in the cells at 72 hours after the virus inoculation.

In addition to the photomicrographs of cell morphological changes, pro-viral load determination indicated that virus copy numbers had increased during the incubation period, as shown in Table 2.

**Table 2:** Virus titer of Vero cells infected with the SARS-CoV-2 virus isolates at a multiplicity of infection (MoI) of 0.04 at 24, 48, and 72 hours post infection.

Incubation period of viral infection	Virus Titer per μL
24 hours	12.10
48 hours	14.29
72 hours	38.19

**Fig 13:**
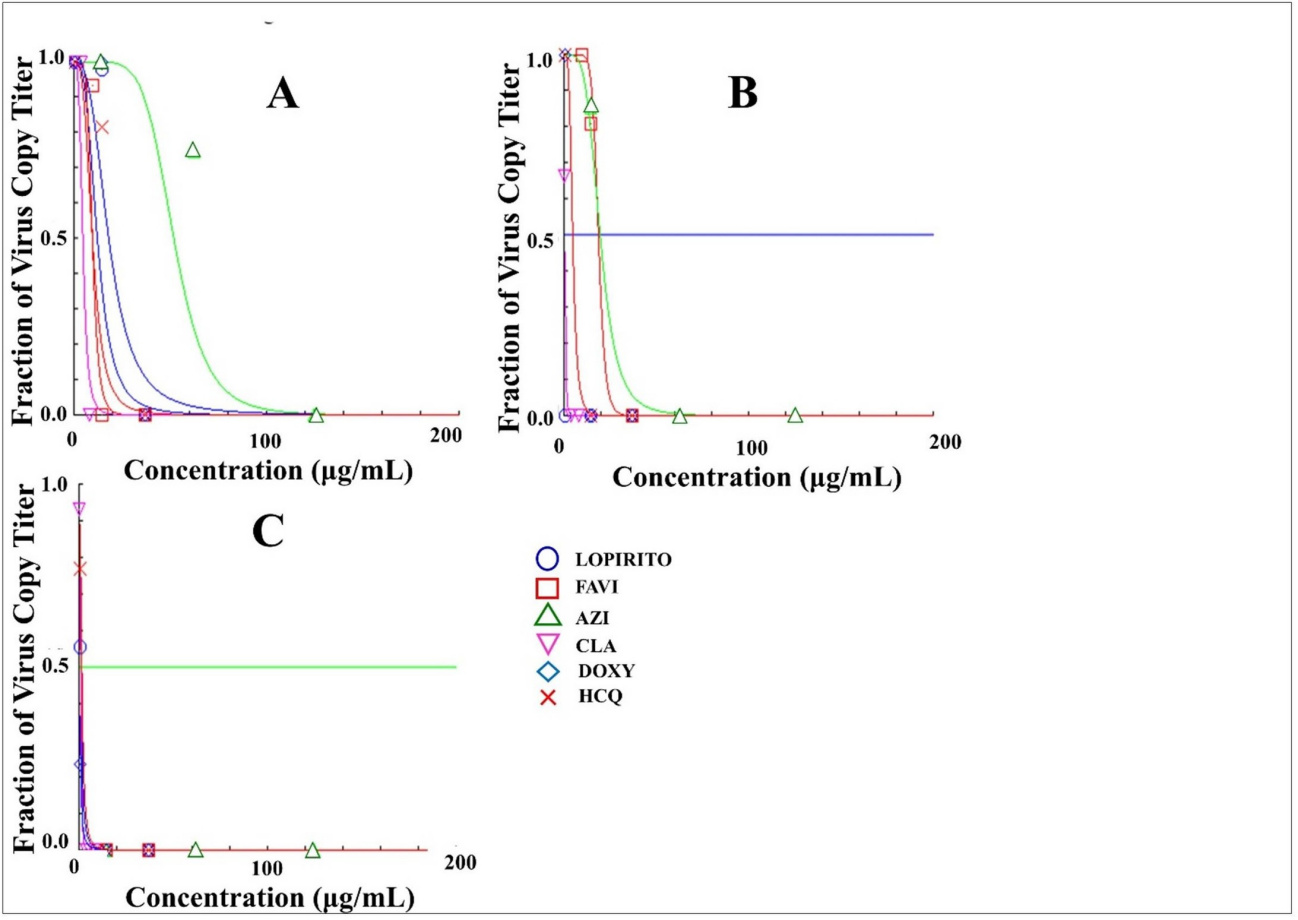
The efficacy (IC_50_) evaluation of Lopinavir-Ritonavir (LOPIRITO), Favipiravir (FAVI), Azithromycin (AZI), Clarithromycin (CLA), Doxycycline (DOXY), and Hydroxychloroquine (HCQ) as a single drug in Vero cells infected with SARS-CoV-2 virus isolates for 24 hours (A), 48 hours (B), and 72 hours (C) analysed using CompuSyn Software at a multiplicity of infection (MoI) value of 0.04.

**Fig 14:**
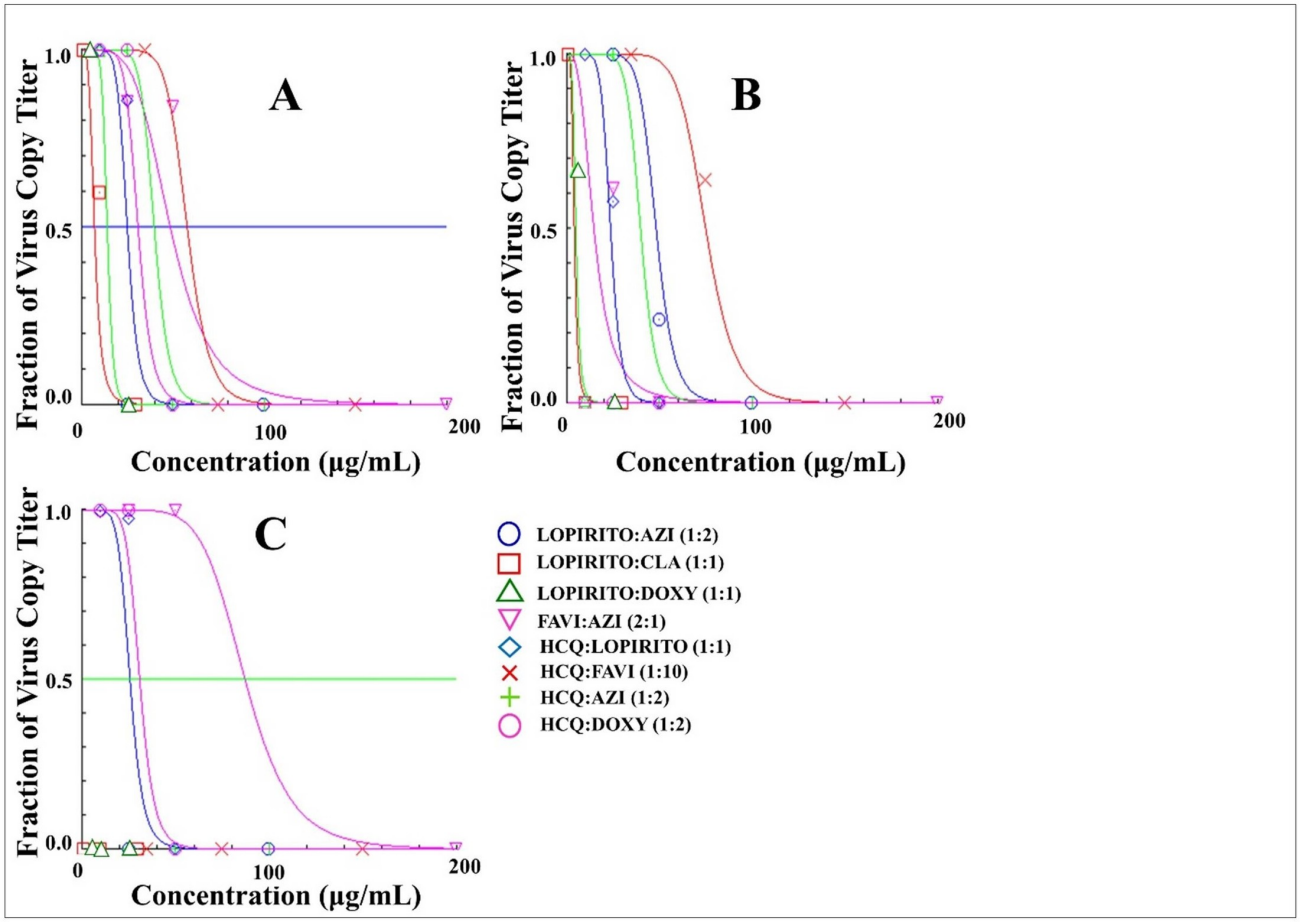
The efficacy (IC_50_) evaluation of dual combination of Lopinavir-Ritonavir (LOPIRITO), Azithromycin (AZI), Doxycycline (DOXY), Favipiravir (FAVI), Clarithromycin (CLA), and Hydroxychloroquine (HCQ) as a single drug in Vero cells infected with SARS-CoV-2 virus isolates for 24 hours (A), 48 hours (B), and 72 hours (C) analysed using CompuSyn Software at a multiplicity of infection (MoI) value of 0.04.

The single drug and dual drug combination were added to the infected Vero cells and incubated for 24, 48 and 72 hours. The virus challenge test (IC_50_ in ppm) of single drug and drug combination against Vero cells infected with SARS-CoV-2 isolate, with a multiplicity of infection (MoI) value of 0.04, showed that combining drugs resulted in lower IC_50_ of each single drug than those of single drug uses. As can be seen in Table 3 and Figs 13 and 14, LOPIRITO + AZI (1:2) resulted in an IC_50_ of less than 8.33 ppm for 24-hour incubation which was lower than those of single use LOPIRITO and AZI which were 12.10 and 51.90 μg/mL respectively. LOPIRITO + CLA (1:1) also produced a similar result at 24 hours post-incubation with a lower IC_50_ value, at 6.90 μg/mL, than those of single LOPIRITO and CLA at 12.10 and 4.60 μg/mL. A drug combination of LOPIRITO + DOXY (1:1) lowered the IC_50_ of DOXY at 24 hours after drug incubation, which was reduced from 18 μg/mL as a single drug to 13.94 μg/mL as a dual drug combination. On the other hand, the combination of HCQ with AZI, DOXY, FAVI, and LOPIRITO increased the IC_50_ values against their single drug uses, as well as the combination of FAVI + AZI (2:1).

**Table 3:** The summary of antiviral activity (IC_50_) of single and combination drugs against Vero cells infected with SARS-CoV-2 at an multiplicity of infection (MoI) value of 0.04.

Drugs	IC_50_ (μg/mL)		
	24h	48h	72h
Lopinavir/Ritonavir (LOPIRITO)	12.10	<1.00	0.90
Azithromycin (AZI)	51.90	19.60	<10.00
Clarithromycin (CLA)	4.60	0.60	0.90
Doxycycline (DOXY)	18.00	4.70	0.40
Hydroxychloroquine (HCQ)	9.50	4.70	1.40
Favipiravir (FAVI)	9.60	18.60	<10.00
Lopinavir/Ritonavir + Azithromycin (LOPIRITO:AZI, 1:2)	<8.33	48.09	<8.33
Lopinavir/Ritonavir + Clarithromycin (LOPIRITO:CLA, 1:1)	6.90	3.90	<0.50
Lopinavir/Ritonavir + Doxycycline (LOPIRITO:DOXY, 1:1)	13.94	4.79	<2.50
Hydroxychloroquine + Azithromycin (HCQ:AZI, 1:2)	39.68	39.68	<16.66
Hydroxychloroquine + Doxycycline (HCQ:DOXY, 1:2)	30.80	<6.67	30.80
Favipiravir + Azithromycin (FAVI:AZI, 2:1)	48.46	14.53	86.99
Hydroxychloroquine + Favipiravir (HCQ:FAVI, 1:10)	57.72	74.77	<31.82
Hydroxychloroquine + Lopinavir/Ritonavir (HCQ:LOPIRITO, 1:2)	24.90	23.49	25.61

On the other hand, the evaluation of each concentration of drug combination at a determined drug incubation period reveals that the use of drug combinations resulted in a lower drug concentration required for producing undetected virus numbers than the single drug uses, as evident from Table 4. The combination of LOPIRITO + AZI (1:2) composed of 13.4 μg/mL LOPIRITO and 33.6 μg/mL AZI had produced undetected virus numbers at 24, 48, and 72 hours post-incubation at a concentration of 50 μg/mL which were lower than the concentrations of each single drug required for generating a similar result, namely; 37.5 and 125 μg/mL for LOPIRITO and AZI respectively. This was also observed for a drug combination of LOPIRITO + CLA(1:1), LOPIRITO + DOXY (1:1), and HCQ + LOPIRITO (1:2). However, the combination of HCQ + AZI (1:2), HCQ + DOXY (1:2), FAVI + AZI (2:1), and HCQ + FAVI (1:10) produced no higher efficacy in respect of virus eradication than their single drugs.

**Table 4:** The concentration of single and combination drugs (at a mass ratio) that produced an undetected virus copy number in the in vitro antiviral study against Vero cells infected with SARS-CoV-2 at a multiplicity of infection (MoI) value of 0.04 at 24, 48, and/or 72 hours’ incubation.

Drugs	Drug concentration (μg/mL)	Results
Lopinavir/Ritonavir (LOPIRITO)	37.5	24, 48, 72h virus undetected
Azithromycin (AZI)	125	24, 48, 72h virus undetected
Clarithromycin (CLA)	8	24, 48, 72h virus undetected
Doxycycline (DOXY)	37.5	24, 48, 72h virus undetected
Hydroxychloroquine (HCQ)	37.5	48, 72h virus undetected
Favipiravir (FAVI)	37.5	24, 48, 72h virus still detected with decreasing number
Lopinavir/Ritonavir + Azithromycin (LOPIRITO:AZI, 1:2)	50	24, 48, 72h virus undetected
Lopinavir/Ritonavir + Clarithromycin (LOPIRITO:CLA, 1:1)	30	48, 72h virus undetected
Lopinavir/Ritonavir + Doxycycline (LOPIRITO:DOXY, 1:1)	25	24, 48, 72h virus undetected
Hydroxychloroquine + Azithromycin (HCQ:AZI, 1:2)	100	24, 48, 72h virus undetected
Hydroxychloroquine + Doxycycline (HCQ:DOXY, 1:2)	25	48, 72h virus undetected
Favipiravir + Azithromycin (FAVI:AZI, 2:1)	200	24, 48, 72h virus still detected with decreasing number
Hydroxychloroquine + Favipiravir (HCQ:FAVI, 1:10)	150	24, 48, 72h virus undetected
Hydroxychloroquine + Lopinavir/Ritonavir (HCQ:LOPIRITO, 1:2)	50	24, 48, 72h virus still detected with decreasing number

### IL-6, IL-10 and TNF-α levels of virus-infected Vero cells incubated with dual combinatory drugs

An analysis of pro-inflammatory and anti-inflammatory responses was further conducted included Interleukin-10 (IL-10), Interleukin-6 (IL-6), and Tumor Necrosis Factor-α (TNF-α). As shown in Table 5, the administration of LOPIRITO, AZI, CLA, and HCQ increased IL-10 levels and reduced the efficacy of IL-6 as a pro-inflammatory marker, but had no effects on TNF-α levels. However, for the most part, the use of dual drug administration increased IL-10 levels as an anti-inflammatory marker and reduced IL-6 and TNF-α levels as pro-inflammatory markers, but there were no noticeable effects on these interleukin levels for the FAVI + AZI (2:1) combination.

**Table 5:** The summary of the cytokine levels of Vero cells infected with SARS-CoV-2 isolates an multiplicity of infection (MoI) value of 0.04 at 24, 48, and 72 hours incubated with single and drug combinations. The data were in duplicates.

Drugs	IL-10	IL-6	TNF-α
Lopinavir/Ritonavir (LOPIRITO)	↗↗ (37.5 μg/mL; 72h)	↘↘ (15 μg/mL; 24, 48h)	No effects
Azithromycin (AZI)	↗↗ (15 μg/mL; 24h)	↘↘ (to 125 μg/mL; 24, 48, 72h)	No effects
Clarithromycin (CLA)	↗↗ (8 μg/mL; 48h)	↘↘ (1, 4, 8 μg/mL; 24, 48, 72h)	No effects
Doxycycline (DOXY)	↗↗ (1 μg/mL; 48, 72h)	↘↘ (1 μg/mL; 24h)	↘↘ (1 μg/mL; 24h)
Hydroxychloroquine (HCQ)	↗↗ (15 μg/mL; 48h)	↘↘ (1 μg/mL; 24h)	No effects
Favipiravir (FAVI)	↗↗ (10, 15 μg/mL; 48, 72h)	↘↘ (to 100 μg/mL; 48h)	↘↘ (10 ppm; 24h)
Lopinavir/Ritonavir + Azithromycin (LOPIRITO:AZI, 1:2)	↗↗ (25, 50, 100 μg/mL; 48,72h) → strong	↘↘ (and IL-2) (25, 50, 100 μg/mL; 24, 48, 72h) → strong IL-2: ↘↘ (100 μg/mL; 24, 48h)	↘↘ (25 ppm; 24h)
Lopinavir/Ritonavir + Clarithromycin (LOPIRITO:CLA, 1:1)	↗↗ (1, 10 μg/mL; 24, 48, 72h)	↘↘ (1 μg/mL; 24, 48h)	↘↘ (30 μg/mL; 24, 48, 72h)
Lopinavir/Ritonavir + Doxycycline (LOPIRITO:DOXY, 1:1)	↗↗ (5, 10 μg/mL; 48, 72h)	↘↘ (and IL-2) (10, 25 μg/mL; 48h) → strong IL-2: ↘↘ (5, 10 μg/mL; 48, 72 h)	↘↘ (5, 10, 25 μg/mL; 24, 48, 72h) → strong
Hydroxychloroquine + Azithromycin (HCQ:AZI, 1:2)	↗↗ (25,50 μg/mL; 48,72h)	↘↘ (and IL-2) (25, 50, 100 μg/mL; 24, 48, 72h) → strong	↘↘ (25 μg/mL; 24h)
Hydroxychloroquine + Doxycycline (HCQ:DOXY, 1:2)	↗↗ (25 μg/mL; 24, 48, 72h)	No effects	↘↘ (10, 25, 50 μg/mL; 24, 48, 72h)
Favipiravir + Azithromycin (FAVI:AZI, 2:1)	No effects	No effects	No effects
Hydroxychloroquine + Favipiravir (HCQ:FAVI, 1:10)	No effects	↘↘ (35, 75 μg/mL; 24h)	No effects
Hydroxychloroquine + Lopinavir/Ritonavir (HCQ:LOPIRITO, 1:2)	↗↗ (25, 50 μg/mL; 48h)	↘↘ (25, 50 μg/mL; 48h)	No effects

**Note:** (25, 50 μg/mL; 48h) means that at concentration of 25 and 50 μg/mL of drug combination, the changes in interleukin levels were observed at48 hours post incubation. ↗↗: increased, ↘↘: decreased.

### Molecular docking study of drugs against main protease of SARS-CoV-2 virus

**Fig 15:**
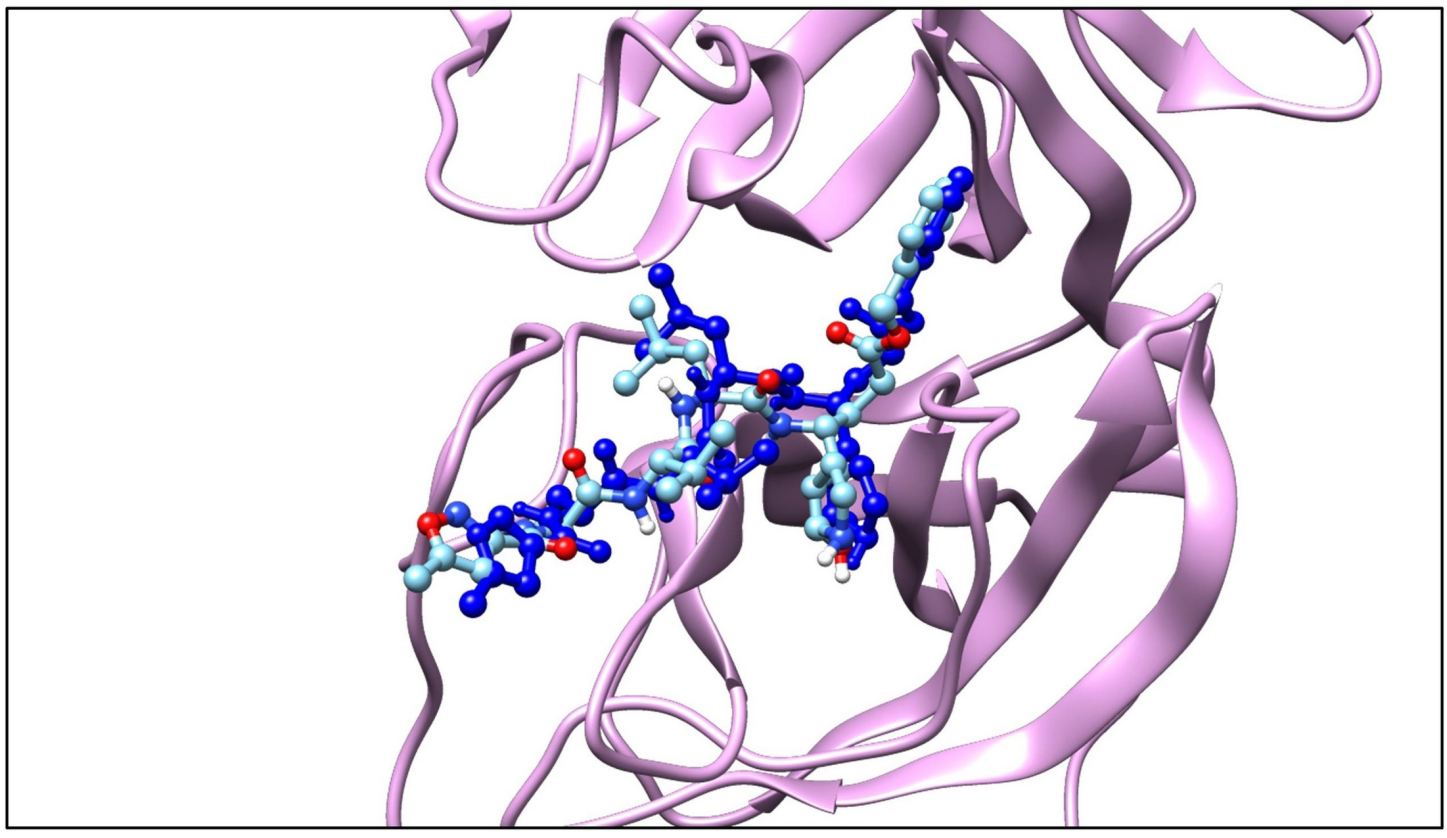
The molecular structures of native ligand binding to receptor in SARS-CoV-2.

By using an in silico method as shown in Fig 15, it can be seen that all the ligands including LOPIRITO, FAVI, AZI, CLA, DOXY, and HCQ can interact with the virus main protease with high docking scores ranging from -37.46 to -22.01 (see Table 6). DOXY recorded the lowest docking score, -37.46 kcal/mol and had a potency higher than Ritonavir (RITO). In contrast, AZI had the highest docking score of approximately -22.01 kcal/mol.

**Table 6:** The docking scores of potential SARS-CoV-2 main protease inhibitor drug.

No	Chemical Name	Molecular Weight (g/mol)	Docking Score (kcal/mol)
1	Lopinavir (LOPI, C_37_H_48_N_4_O_5_)	628.8	-28.56
2	Ritonavir (RITO, C_37_H_48_N_6_O_5_S_2_)	720.9	-30.47
3	Favipiravir (FAVI, C_5_H_4_FN_3_O_2_)	157.1	-23.11
4	Azithromycin (AZI, C_38_H_72_N_2_O_12_)	749	-22.01
5	Clarithromycin (CLA, C_38_H_69_NO_13_)	748	-25.48
6	Doxycycline (DOXY, C_22_H_24_N_2_O_8_)	444.4	-37.46
7	Hydroxychloroquine (HCQ, C_18_H_26_ClN_3_O)	335.9	-29.59

The parameters to validate the docking parameters were employed to perform the docking of each candidate ligand. From the docking results, the binding energy was obtained in the form of a grid score (kcal / mol) for each ligand to the receptor as presented in Table 6.

## Discussion

The in vitro antiviral activities of dual combinatory drugs consisting of antiviral agents, i.e. LOPIRITO, FAVI, antibiotics such as AZI, CLA, DOXY, and HCQ against Vero cells infected with SARS-CoV-2 virus isolated from hospitalized patients in Surabaya, Indonesia were evaluated. These drugs have recently became the subject of interest for use in clinical trials, thereby providing information about their therapeutic effects as combinatory drugs within a highly effective strategy of providing pre-clinical evidence supporting their clinical use for combating pandemic COVID-19.

LOPIRITO is a protease inhibitor commonly employed in the treatment of HIV that, interestingly, has also been shown to have an antiviral effect on SARS-CoV and MERS-CoV by inhibiting the protease activity of coronavirus [17, 18, 32]. Within this study, its combined use with other drugs was evaluated. Significantly, most of these drug combinations demonstrated greater in vitro antiviral potency against the SARS-CoV-2 virus with lower cytotoxicity observed in mesenchymal stem cells than the single drug itself.

The drug combinations were prepared in two ratio types, i.e. constant and non-constant weight ratios, due to the lack of data regarding the growth inhibition curves of these drugs in mesenchymal stem cells in addition to their IC_50_ values. Moreover, there is a paucity of information about which drug is more toxic to the cells and drug use in combination as evaluated in this study. This study aimed to identify the profile of drug interaction, whether synergistic, additional, or antagonistic, in order to establish their cytotoxic effect on mesenchymal stem cells. In principal, to obtain the appropriate ratio for clinical use, drug combinations were prepared at both constant and non-constant ratios, with their IC50 values being subsequently determined. After the profiles had been obtained, the constant ratio with low cytotoxicity was selected for further antiviral evaluation, while the non-constant ratio was not considered further. This was because the use of commercial products at a largely general dosage represents a more practical therapeutic application of COVID-19, not involving a customized dose or Fixed Dose Combination products.

LOPIRITO was combined with AZI, primarily used in the treatment of respiratory, enteric and genitourinary infection, which had also been recently employed as a therapeutic agent against COVID-19 infection [33]. In this study, the dual combination of LOPIRITO and AZI at respective ratios of 1:1 and 1:2 reduced the cytotoxicity of each single drug on mesenchymal stem cells. Moreover, their combination produced higher efficacy in reducing virus numbers, while also increasing IL-10 and reducing IL-6 and TNF-α levels.

LOPIRITO was also combined with CLA. Instead of monotherapy using only LOPIRITO, several hospitalized patients received CLA, a macrolide antibiotic, which inhibits protein synthesis in susceptible organisms (e.g. bacteria) by binding to the 50S ribosomal sub-unit [34]. The same results were also achieved by combining LOPIRITO and CLA at a weight ratio of 1:1. There was a decrease in cytotoxicity in normal cells and an increase of antiviral activity against SARS-CoV-2 virus compared with each single drug.

FAVI is an antiviral medication used to treat influenza in Japan which is also being evaluated for its effectiveness against other viral infections [35]. However, there is evidence that FAVI is teratogenic, with the result that considerable care needs to be exercised in avoiding its extensive use during pregnancy [36, 37]. AZI is a broad-spectrum macrolide antibiotic with a long half-life, excellent tissue penetration and a large distribution volume [9, 21]. DOXY is a broad-spectrum tetracycline-class antibiotic used in the treatment of infections caused by bacteria and certain parasites. It is used to treat bacterial pneumonia, acne, chlamydia infections, early-stage Lyme disease, cholera, typhus, and syphilis [38]. HCQ is a medication used to prevent and treat malaria in areas where the disease remains resistant to chloroquine. Other applications include the treatment of rheumatoid arthritis, lupus, and porphyria cutanea tarda. HCQ is currently being studied to establish its efficacy in the prevention and treatment of COVID-19 [39].

The same results are also obtained by use of a combination of LOPIRITO + CLA (Fig 5), LOPIRITO + DOXY (Fig 6), HCQ + AZI (Fig 7), and HCQ + DOXY (Fig 8). These combinations showed the absence of cytotoxic effect in cells and viability exceeding 90%. The use of this combination provides a potential opportunity for antiviral testing due to its minimal toxic effects on mesenchymal cells.

Both FAVI and AZI, when administered as single drugs, and their combination (FAVI + AZI) produce extremely low cytotoxicity since they are relatively non-toxic to mesenchymal cells, as indicated by the high CC_50_ value, (see Fig 9). On the other hand, a drug combination of FAVI + HCQ has a higher CC_50_ value than HCQ as a single drug, which is relatively more toxic than FAVI, as can be seen from the contents of Fig 10. A combination of LOPIRITO + HCQ also has a higher CC_50_ value than HCQ as a single drug which is relatively more toxic than LOPIRITO, (see Fig 11).

Based on the CC_50_ value data obtained, the application of a combination of LOPIRITO, AZI, CLA, DOXY, FAVI, and HCQ has the potential to reduce the degree of toxicity of the drug administered. Most drug combinations exhibit antagonistic effects which negate the side effects of other drugs. Thus, when viewed from the perspective of safety and toxicity, the potential use of a combination of therapeutic drugs, especially the treatment of COVID-19, is extremely high and can be considered effective. Furthermore, a virus challenge test was performed on a combination of drugs which was declared to be relatively safe.

Antiviral activity was assessed using Vero cells previously infected with SARS-CoV-2 isolates obtained from Universitas Airlangga Hospital. A summary of results can be seen in Table 3. It can be noted that the use of a single drug has the ability to reduce the amount of virus. The analysis involving the use of software can be seen in Fig 13. With a single drug, there was a decrease in the number of copies of the virus (Fa = number of copies of virus samples / positive controls) in accordance with the duration of drug incubation in the sample, whereby at 72 hours, almost all viruses in the test group had died. The antiviral activities of drug combinations can be seen in Fig 14 with a summary of the results contained in Table 4. The results indicate that drug combinations demonstrated greater effectiveness in reducing the amount of virus where IC_50_ values decreased after 24, 48 and 72 hours of the incubating of cells infected with the drug. As a combination drug, there was a decrease in the number of copies of the virus in some samples whereas, depending on the incubation time of the drug in the sample, there was a significant reduction in the amount of virus in the test group.

An analysis of pro-inflammatory and anti-inflammatory responses was conducted, including Interleukin-10 (IL-10), Interleukin-6 (IL-6), and Tumor Necrosis Factor-α (TNF-α). From the results presented in Table 5, the majority of drug administration increased IL-10 levels as an anti-inflammatory marker and reduced IL-6 and TNF-α levels as pro-inflammatory markers. Only in the combination of FAVI + AZI (2:1) was the effect negligible. The interactions observed in this study can be physical or chemical and affect the ability of the drugs to infiltrate the cell to cause further toxic effects and inhibit or reduce the rate of viral infectivity in host cells.

Molecular docking was employed to predict interactions between ligands and protein. The interaction can be seen from the binding site of the macromolecular target. The docking process consists of two interrelated stages, docking algorithm and scoring function. The docking algorithm obtains the most stable conformation of the ligand-protein complex formed. Molecular bonds will be formed from functional groups of ligands that interact with residues of amino acid receptor proteins. The scoring function is intended to evaluate conformation by calculating the strength of the affinity between ligand and protein and then directing the exploration of the ligand conformation to a position with a stronger affinity [40]. The affinity value obtained was in the form of Gibbs free energy. A low Gibbs free energy value indicates that the conformation formed is stable, while a high one indicates the formation of a less stable complex. The more negative the value produced, the stronger the affinity of the ligand-protein complex, with the result that its activity is expected to be of even higher quality [41, 42].

The SARS-CoV-2 main protease (PDB ID: ALU6) is a ~306 amino acid long main protease whose crystal structure with a resolution of 1.93 Å has been elucidated. The main protease enzyme is the optimum target for inhibiting the SARS-CoV-2 virus. This protease breaks the spikes and is further established by penetration. This study was undertaken to identify possible compounds that can bind to the main protease which may be used as a potential drug for SARS-CoV-2. The results indicated that all the ligands, i.e. LOPI, RITO, FAVI, AZI, CLA, DOXY, and HCQ, can bind with the main protease with a high docking score of -37.46 to -22.01 kcal/mol (see Table 6). It is probable that the compounds inhibit the process of viral replication and translation and may have an extremely significant impact on controlling the viral load in infected individuals.

## Conclusion

Using a combination of drugs would reduce the degree of cytotoxicity compared to a single drug, increase antiviral activity, and produce a lower effect on pro-inflammatory markers and intensify anti-inflammatory response. Hence, it can reduce the toxic potency in cells and increase the effectiveness with regard to reducing the number of copies of the SARS-CoV-2 virus. Based on the degree of therapeutic effectiveness, toxicity in vitro, and response to inflammatory markers, the activity of a single drug from the highest to the lowest is as follows: CLA > LOPIRITO > DOXY > AZI > HCQ.

Based on the degree of therapeutic effectiveness, toxicity in vitro, and the response to inflammatory markers, the activity of a drug combination ranging from the highest to lowest is the following: LOPIRITO + AZI > LOPIRITO + AZI > HCQ + AZI > HCQ + FAVI > LOPIRITO + CLA > HCQ + DOXY. However, further studies are required regarding the possible interactions.

## Supporting information

S1 Table The cytotoxicity data of combinatory drugs on mesenchymal human stem cells. (PDF)

S2 Table The average virus titer of Vero cells infected with SARS-CoV-2 isolates an multiplicity of infection (MoI) value of 0.04 at 24, 48, and 72 hours incubated with single and drug combinations (n = 2). (PDF)

S3 Table The cytokine levels of Vero cells infected with SARS-CoV-2 isolates an multiplicity of infection (MoI) value of 0.04 at 24, 48, and 72 hours incubated with single and drug combinations (n = 2). (PDF)

## References

[1] Huang C, Wang Y, Li X, Ren L, Zhao J, Hu Y, et al. Clinical features of patients infected with 2019 novel coronavirus in Wuhan, China. Lancet. 2020; 395(10223): 497–506. doi: 10.1016/S0140-6736(20)30183-5 31986264 PMC7159299

[2] Lu R, Zhao X, Li J, Niu P, Yang B, Wu H, et al. Genomic characterisation and epidemiology of 2019 novel coronavirus: implications for virus origins and receptor binding. Lancet. 2020; 395(10224): 565–74. doi: 10.1016/S0140-6736(20)30251-8 32007145 PMC7159086

[3] Zhu N, Zhang D, Wang W, Li X, Yang B, Song J, et al. A novel coronavirus from patients with pneumonia in China, 2019. N Engl J Med. 2020; 382: 727–733. doi: 10.1056/NEJMoa2001017 31978945 PMC7092803

[4] Manusubroto W, Wicaksono AS, Tamba DA, Sudiharto P, Pramusinto H, Hartanto RA, et al. Neurosurgery services in Dr. Sardjito General Hospital, Yogyakarta, Indonesia, during COVID-19 pandemic: an experience from a developing country. World Neurosurg. 2020; 140: e360–e366. doi: 10.1016/j.wneu.2020.05.124 32442732 PMC7237373

[5] Dayer MR, Taleb-Gassabi S, Dayer MS. Lopinavir; a potent drug against coronavirus infection: insight from molecular docking study. Arch Clin Infect Dis. 2017; 12(4): e13823.

[6] Dong L, Hu S, Gao J. Discovering drugs to treat coronavirus disease 2019 (COVID-19). Drug Discov Ther. 2020; 14(1): 58–60. doi: 10.5582/ddt.2020.01012 32147628

[7] Jin Y-H, Cai L, Cheng Z-S, Cheng H, Deng T, Fan Y-P, et al. A rapid advice guideline for the diagnosis and treatment of 2019 novel coronavirus (2019-nCoV) infected pneumonia (standard version). Mil Med Res. 2020; 7(1): 4. doi: 10.1186/s40779-020-0233-6 32029004 PMC7003341

[8] AbbVie Deutschland GmbH & Co. KG. AluviaH-W-764: Summary of Product Characteristics. European Medicines Agency, Germany; 2020 [cited 15 March 2021]. Available from: https://www.ema.europa.eu/en/aluvia-h-w-764

[9] Singlas E. [Clinical pharmacokinetics of azithromycin]. Pathol Biol (Paris). 1995 Jun; 43(6): 505–11. 8539072

[10] Fraschini F, Scaglione F, Demartini G. Clarithromycin clinical pharmacokinetics. Drug Dispos. 1993; 25(3): 189–204. doi: 10.2165/00003088-199325030-00003 8222460

[11] Newton PN, Brockman A, Chierakul W, Dondorp A, Ruangveerayuth R, Looareesuwan S, et al. Pharmacokinetics of oral doxycycline during combination treatment of severe falciparum malaria. Antimicrob Agents Chemother. 2005; 49(4): 1622–5. doi: 10.1128/AAC.49.4.1622-1625.2005 15793155 PMC1068593

[12] Lim H, Im J, Cho J, Bae K, Klein TA, Yeom J, et al. Pharmacokinetics of Hydroxychloroquine and its clinical implications in chemoprophylaxis against malaria caused by plasmodium vivax. Antimicrob Agents Chemother. 2009; 53(4): 1468–75. doi: 10.1128/AAC.00339-08 19188392 PMC2663072

[13] Taisho Toyama Pharmaceutical. Avigan Tablets 200 mg. 2017 [cited 15 March 2021]. Available from: https://www.cdc.gov.tw/File/Get/ht8jUiB_MI-aKnlwstwzvw.

[14] Retallack H, Di E, Arias C, Knopp KA, Laurie MT. Zika virus cell tropism in the developing human brain and inhibition by azithromycin. PNAS. 2016; 113(50): 14408–12. doi: 10.1073/pnas.1618029113 27911847 PMC5167169

[15] Dayer MR. Old drugs for newly emerging viral disease, COVID-19: bioinformatic prospective. arXiv:2003.04524 [Preprint]. 2020 [cited 7 March 2021]. Available from: https://arxiv.org/abs/2003.04524

[16] Arabi YM, Deeb AM, Al-hameed F, Mandourah Y, Almekhla GA, Sindi AA, et al. Macrolides in critically ill patients with middle east respiratory syndrome. Int J Infect Dis. 2019; 81: 184–90. doi: 10.1016/j.ijid.2019.01.041 30690213 PMC7110878

[17] Rothan HA, Mohamed Z. Inhibitory effect of doxycycline against dengue virus replication in vitro. Arch Virol. 2014; 159: 711–8. doi: 10.1007/s00705-013-1880-7 24142271

[18] Liu X, Wan X-J. Potential inhibitors against 2019-nCoV coronavirus M protease from clinically approved medicines. J Genet Genomics. 2020; 47: 119–21. doi: 10.1016/j.jgg.2020.02.001 32173287 PMC7128649

[19] Bacharier LB, Guilbert TW, Mauger DT, Boehmer S, Beigelman A, Fitzpatrick AM, et al. Early administration of azithromycin and prevention of severe lower respiratory tract illnesses in preschool children with a history of such illnesses: a randomized clinical trial. JAMA. 2016; 314(19): 2034–44. 10.1001/jama.2015.13896 PMC4757487 26575060

[20] Sahraei Z, Shabani M, Shokouhi S, Saffaei A. Aminoquinolines against coronavirus disease 2019 (COVID-19): chloroquine or hydroxychloroquine. Int J Antimicrob Agents. 2020; 2020: 55(4): 105945. doi: 10.1016/j.ijantimicag.2020.105945 32194152 PMC7156117

[21] Devaux CA, Rolain J, Colson P, Raoult D. New insights on the antiviral effects of chloroquine against coronavirus: what to expect for COVID-19? Int J Antimicrob Agents. 2020; 55(5): 105938. doi: 10.1016/j.ijantimicag.2020.105938 32171740 PMC7118659

[22] Savarino A, Boelaert JR, Cassone A, Majori G, Cauda R. Antiviral effects of chloroquine: Effects of chloroquine on viral infections: an old drug against today’s diseases? Lancet Infect Dis. 2003; 3(11): 722–7. doi: 10.1016/s1473-3099(03)00806-5 14592603 PMC7128816

[23] Sargiacomo C, Sotgia F, Lisanti MP. COVID-19 and chronological aging: senolytics and other anti-aging drugs for the treatment or prevention of corona virus infection? Aging (Albany NY). 2020;12(8): 6511–6517. doi: 10.18632/aging.103001 32229706 PMC7202514

[24] Chu CM, Cheng VCC, Hung IFN, Wong MML, Chan KH, Chan KS, et al. Role of lopinavir/ritonavir in the treatment of SARS: initial virological and clinical findings. Thorax. 2004; 59(3): 252–6. doi: 10.1136/thorax.2003.012658 14985565 PMC1746980

[25] Jin Z, Du X, Xu Y, Deng Y, Liu M, Zhao Y, et al. Structure of Mpro from SARS-CoV-2 and discovery of its inhibitors. Nature. 2020; 582(7811): 289–93. doi: 10.1038/s41586-020-2223-y 32272481

[26] Wang Z, Sun H, Yao X, Li D, Xu L, Li Y, et al. Comprehensive evaluation of ten docking programs on a diverse set of protein-ligand complexes: The prediction accuracy of sampling power and scoring power. Phys Chem Chem Phys. 2016; 18(18): 12964–75. doi: 10.1039/c6cp01555g 27108770

[27] Maier JA, Martinez C, Kasavajhala K, Wickstrom L, Hauser KE, Simmerling C. ff14SB: Improving the Accuracy of Protein Side Chain and Backbone Parameters from ff99SB. J Chem Theory Comput. 2015; 11(8): 3696–713. doi: 10.1021/acs.jctc.5b00255 26574453 PMC4821407

[28] Allen WJ, Balius TE, Mukherjee S, Brozell SR, Moustakas DT, Lang PT, et al. DOCK 6: Impact of new features and current docking performance. J Comput Chem. 2015; 36(15): 1132–56. doi: 10.1002/jcc.23905 25914306 PMC4469538

[29] Covas D, Siufi J, Silva A, Orellana M. Isolation and culture of umbilical vein mesenchymal stem cells. Brazilian J Med Biol Res. 2003; 36(9): 1179–83. doi: 10.1590/s0100-879x2003000900006 12937783

[30] Lu L, Liu Y-J, Yang S-G, Zhao Q, Wang X, Gong W, et al. Isolation and characterization of human umbilical cord mesenchymal stem cells with hematopoiesis-supportive function and other potentials. Haematologica. 2006; 91(8): 1017–26. 16870554

[31] Mennan C, Wright K, Bhattacharjee A, Balain B, Richardson J, Roberts S. Isolation and characterisation of mesenchymal stem cells from different regions of the human umbilical cord. Biomed Res Int. 2013; 2013: 1–8. doi: 10.1155/2013/916136 23984420 PMC3741948

[32] Chan JF-W, Yao Y, Yeung M-L, Deng W, Bao L, Jia L, et al. Treatment with lopinavir/ritonavir or interferon-β1b improves outcome of MERS-CoV infection in a nonhuman primate model of common marmoset. J Infect Dis. 2015; 212(12): 1904–13. doi: 10.1093/infdis/jiv392 26198719 PMC7107395

[33] Damle B, Vourvahis M, Wang E, Leaney J, Corrigan B. Clinical pharmacology perspectives on the antiviral activity of azithromycin and use in COVID‐19. Clin Pharmacol Ther. 2020; 108(2): 201–211. doi: 10.1002/cpt.1857 32302411 PMC7262099

[34] Rosenberg ES, Dufort EM, Udo T, Wilberschied LA, Kumar J, Tesoriero J, et al. Association of treatment with hydroxychloroquine or azithromycin with in-hospital mortality in patients with COVID-19 in New York state. JAMA. 2020; 323(24): 2493–2502. doi: 10.1001/jama.2020.8630 32392282 PMC7215635

[35] Dinos GP. The macrolide antibiotic renaissance. Br J Pharmacol. 2017; 174: 2967–83. doi: 10.1111/bph.13936 28664582 PMC5573421

[36] Du Y, Chen X. Favipiravir: pharmacokinetics and concerns about clinical trials for 2019‐nCoV infection. Clin Pharmacol Ther. 2020; 108(2): 242–247. doi: 10.1002/cpt.1844 32246834

[37] Agrawal U, Raju R, Udwadia ZF. Favipiravir: A new and emerging antiviral option in COVID-19. Med J Armed Forces India. 2020; 76(4):370–376. doi: 10.1016/j.mjafi.2020.08.004 32895599 PMC7467067

[38] Bacharier LB, Guilbert TW, Mauger DT, Boehmer S, Beigelman A, Fitzpatrick AM, et al. Early administration of azithromycin and prevention of severe lower respiratory tract illnesses in preschool children with a history of such illnesses: a randomized clinical trial. JAMA. 2015; 314(19): 2034–44. doi: 10.1001/jama.2015.13896 26575060 PMC4757487

[39] Nelson ML, Levy SB. The history of the tetracyclines. Ann N Y Acad Sci. 2011; 1241: 17–32. doi: 10.1111/j.1749-6632.2011.06354.x 22191524

[40] Meyerowitz EA, Vannier AGL, Friesen MGN, Schoenfeld S, Gelfand JA, Callahan M V, et al. Rethinking the role of hydroxychloroquine in the treatment of COVID‐19. FASEB J. 2020; 34(5): 6027–37. doi: 10.1096/fj.202000919 32350928 PMC7267640

[41] Meng X-Y, Zhang H-X, Mezei M, Cui M. Molecular docking: A powerful approach for structure-based drug discovery. Curr Comput Aided Drug Des. 2011; 7(2): 146–57. doi: 10.2174/157340911795677602 21534921 PMC3151162

[42] Du X, Li Y, Xia Y, Ai S, Liang J, Sang P, et al. Insights into protein–ligand interactions: mechanisms, models, and methods. Int J Mol Sci. 2016; 17(144): 1–34. 10.3390/ijms17020144 PMC4783878 26821017

