# Supplementary material for: An in vitro study of dual drug combinations of anti-viral agents, antibiotics, and/or hydroxychloroquine against the SARS-CoV-2 virus isolated from hospitalized patients in Surabaya, Indonesia
Source: PLoS One. 2021 Jun 18;16(6):e0252302. doi: 10.1371/journal.pone.0252302 (PMC8213153; doi:10.1371/journal.pone.0252302)
Supplement: S1 Table — (PDF) [file pone.0252302.s001.pdf]

S1 Table. The Cytotoxicity Data of combinatory drugs on mesenchymal human stem cells

LOPIRITO-AZITHROMYCIN

LOPIRITO (as LOPINAVIR in µg/mL)							
**No**	**Control (Media)**	**Control (Cell)**	**0.2**	**2**	**10**	**100**	**400**
1	0.092	0.316	0.372	0.332	0.285	0.267	0.268
2	0.085	0.344	0.386	0.338	0.283	0.254	0.278
3	0.092	0.349	0.383	0.332	0.283	0.264	0.284
Total	0.269	1.009	1.141	1.002	0.851	0.785	0.830
Mean	0.090	0.336	0.380	0.334	0.284	0.262	0.277
Viability Cell (%)			117.8	99.1	78.7	69.7	75.8

AZITHROMYCIN (in µg/mL)							
**No**	**Control (Media)**	**Control (Cell)**	**0.2**	**2**	**10**	**100**	**400**
1	0.092	0.316	0.285	0.289	0.298	0.292	0.270
2	0.085	0.344	0.305	0.292	0.291	0.321	0.280
3	0.092	0.349	0.306	0.289	0.306	0.318	0.320
Total	0.269	1.009	0.896	0.870	0.895	0.931	0.870
Mean	0.090	0.336	0.299	0.290	0.298	0.310	0.290
Viability Cell (%)			84.7	81.2	84.6	89.5	81.2

LOPIRITO-AZI COMBINATIONS (1/1)							
**No**	**Control (Media)**	**Control (Cell)**	**0.2**	**2**	**10**	**100**	**400**
1	0.092	0.316	0.280	0.313	0.296	0.246	0.260
2	0.085	0.344	0.291	0.317	0.300	0.255	0.273
3	0.092	0.349	0.292	0.318	0.296	0.295	0.280
Total	0.269	1.009	0.863	0.948	0.892	0.796	0.813
Mean	0.090	0.336	0.288	0.316	0.297	0.265	0.271
Viability Cell (%)			80.3	91.8	84.2	71.2	73.5

LOPIRITO-AZI COMBINATIONS (1/2)							
**No**	**Control (Media)**	**Control (Cell)**	**0.2**	**2**	**10**	**100**	**400**
1	0.092	0.316	0.286	0.306	0.301	0.325	0.210
2	0.085	0.344	0.305	0.321	0.318	0.354	0.216
3	0.092	0.349	0.305	0.314	0.295	0.304	0.220
Total	0.269	1.009	0.896	0.941	0.914	0.983	0.646
Mean	0.090	0.336	0.299	0.314	0.305	0.328	0.215
Viability Cell (%)			84.7	90.8	87.2	96.5	51.0

LOPIRITO 8 µg/mL + AZI (in µg/mL)							
**No**	**Control (Media)**	**Control (Cell)**	**0.2**	**2**	**10**	**100**	**400**
1	0.092	0.316	0.293	0.303	0.305	0.303	0.263
2	0.085	0.344	0.293	0.299	0.308	0.303	0.265
3	0.092	0.349	0.287	0.336	0.322	0.306	0.280
Total	0.269	1.009	0.873	0.938	0.935	0.912	0.808
Mean	0.090	0.336	0.291	0.313	0.312	0.304	0.269
Viability Cell (%)			81.6	90.4	90.0	86.9	72.8

LOPIRITO (in µg/mL) + AZI 50 µg/mL							
**No**	**Control (Media)**	**Control (Cell)**	**0.2**	**2**	**10**	**100**	**400**
1	0.092	0.316	0.354	0.347	0.375	0.373	0.352

2	0.085	0.344	0.355	0.335	0.390	0.349	0.362
3	0.092	0.349	0.340	0.333	0.387	0.329	0.329
Total	0.269	1.009	1.049	1.015	1.152	1.051	1.043
Mean	0.090	0.336	0.350	0.338	0.384	0.350	0.348
Viability Cell (%)			105.4	100.8	119.3	105.7	104.6

LOPIRITO-CLARITHROMYCIN

LOPIRITO (as LOPINAVIR in µg/mL)							
**No**	**Control (Media)**	**Control (Cell)**	**0.2**	**2**	**10**	**100**	**400**
1	0.092	0.316	0.326	0.302	0.234	0.260	0.229
2	0.085	0.318	0.309	0.311	0.261	0.251	0.266
3	0.092	0.328	0.319	0.300	0.258	0.265	0.259
Total	0.269	0.962	0.954	0.913	0.753	0.776	0.754
Mean	0.090	0.321	0.318	0.304	0.251	0.259	0.251
Cell Viability (%)			98.8	92.9	69.9	73.2	70.0

CLARITHROMYCIN (in µg/mL)							
**No**	**Control (Media)**	**Control (Cell)**	**0.2**	**2**	**10**	**100**	**400**
1	0.092	0.316	0.388	0.285	0.305	0.285	0.266
2	0.085	0.318	0.408	0.314	0.266	0.314	0.263
3	0.092	0.328	0.405	0.312	0.248	0.312	0.255
Total	0.269	0.962	1.201	0.911	0.819	0.911	0.784
Mean	0.090	0.321	0.400	0.304	0.273	0.304	0.261
Cell Viability (%)			134.5	92.6	79.4	92.6	74.3

LOPIRITO-CLA COMBINATIONS (1/1)							
**No**	**Control (Media)**	**Control (Cell)**	**0.2**	**2**	**10**	**100**	**400**
1	0.092	0.316	0.260	0.321	0.262	0.262	0.260
2	0.085	0.318	0.302	0.340	0.266	0.248	0.237
3	0.092	0.328	0.307	0.351	0.253	0.272	0.268
Total	0.269	0.962	0.869	1.012	0.781	0.782	0.765
Mean	0.090	0.321	0.290	0.337	0.260	0.261	0.255
Cell Viability (%)			86.6	107.2	73.9	74.0	71.6

LOPIRITO-CLA COMBINATIONS (1/0.5)							
**No**	**Control (Media)**	**Control (Cell)**	**0.2**	**2**	**10**	**100**	**400**
1	0.092	0.316	0.423	0.400	0.312	0.248	0.242
2	0.085	0.318	0.437	0.335	0.305	0.238	0.291
3	0.092	0.328	0.400	0.338	0.287	0.306	0.271
Total	0.269	0.962	1.260	1.073	0.904	0.792	0.804
Mean	0.090	0.321	0.420	0.358	0.301	0.264	0.268
Cell Viability (%)			143.0	116.0	91.6	75.5	77.2

LOPIRITO 8 µg/mL + CLA (in µg/mL)							
**No**	**Control (Media)**	**Control (Cell)**	**0.2**	**2**	**10**	**100**	**400**
1	0.092	0.316	0.317	0.299	0.280	0.299	0.275
2	0.085	0.318	0.285	0.268	0.282	0.268	0.270
3	0.092	0.328	0.300	0.262	0.270	0.262	0.264

Total	0.269	0.962	0.902	0.829	0.832	0.829	0.809
Mean	0.090	0.321	0.301	0.276	0.277	0.276	0.270
Cell Viability (%)			91.3	80.8	81.2	80.8	77.9

LOPIRITO (in µg/mL) + CLA 1 µg/mL							
**No**	**Control (Media)**	**Control (Cell)**	**0.2**	**2**	**10**	**100**	**400**
1	0.092	0.316	0.389	0.315	0.358	0.315	0.345
2	0.085	0.318	0.375	0.307	0.327	0.307	0.318
3	0.092	0.328	0.377	0.360	0.336	0.360	0.325
Total	0.269	0.962	1.141	0.982	1.021	0.982	0.988
Mean	0.090	0.321	0.380	0.327	0.340	0.327	0.329
Cell Viability (%)			125.8	102.9	108.5	102.9	103.8

LOPIRITO-DOXYCYCLINE

LOPIRITO (as LOPINAVIR in µg/mL)							
**No**	**Control (Media)**	**Control (Cell)**	**0.2**	**2**	**10**	**100**	**400**
1	0.094	0.387	0.536	0.480	0.413	0.344	0.253
2	0.092	0.366	0.530	0.452	0.399	0.380	0.296
3	0.100	0.392	0.532	0.459	0.399	0.374	0.321
Total	0.286	1.145	1.598	1.391	1.211	1.098	0.870
Mean	0.095	0.382	0.533	0.464	0.404	0.366	0.290
Cell Viability (%)			152.7	128.6	107.7	94.5	68.0

DOXYCYCLINE (µg/mL)							
**No**	**Control (Media)**	**Control (Cell)**	**0.2**	**2**	**10**	**100**	**400**
1	0.094	0.387	0.389	0.387	0.397	0.352	0.355
2	0.092	0.366	0.390	0.390	0.397	0.371	0.346
3	0.100	0.392	0.392	0.406	0.394	0.360	0.373
Total	0.286	1.145	1.171	1.183	1.188	1.083	1.074
Mean	0.095	0.382	0.390	0.394	0.396	0.361	0.358
Cell Viability (%)			103.0	104.4	105.0	92.8	91.7

LOPIRITO-DOXY COMBINATIONS (1/1)							
**No**	**Control (Media)**	**Control (Cell)**	**0.2**	**2**	**10**	**100**	**400**
1	0.094	0.387	0.441	0.413	0.380	0.413	0.374
2	0.092	0.366	0.442	0.416	0.378	0.416	0.387
3	0.100	0.392	0.430	0.405	0.392	0.405	0.383
Total	0.286	1.145	1.313	1.234	1.150	1.234	1.144
Mean	0.095	0.382	0.438	0.411	0.383	0.411	0.381
Cell Viability (%)			119.6	110.4	100.6	110.4	99.9

LOPIRITO-DOXY COMBINATIONS (1/2)							
**No**	**Control (Media)**	**Control (Cell)**	**0.2**	**2**	**10**	**100**	**400**
1	0.094	0.387	0.423	0.416	0.400	0.406	0.407
2	0.092	0.366	0.443	0.432	0.425	0.420	0.397
3	0.100	0.392	0.493	0.398	0.412	0.397	0.411
Total	0.286	1.145	1.359	1.246	1.237	1.223	1.215
Mean	0.095	0.382	0.453	0.415	0.412	0.408	0.405

Cell Viability (%)			124.9	111.8	110.7	109.1	108.1

LOPIRITO (μg/mL) + DOXY 2 μg/mL							
**No**	**Control (Media)**	**Control (Cell)**	**0.2**	**2**	**10**	**100**	**400**
1	0.094	0.387	0.515	0.441	0.390	0.377	0.389
2	0.092	0.366	0.492	0.460	0.386	0.381	0.370
3	0.100	0.392	0.495	0.453	0.400	0.393	0.374
Total	0.286	1.145	1.502	1.354	1.176	1.151	1.133
Mean	0.095	0.382	0.501	0.451	0.392	0.384	0.378
Cell Viability (%)			141.6	124.3	103.6	100.7	98.6

LOPIRITO 8 μg/mL + DOXY (in μg/mL)							
**No**	**Control (Media)**	**Control (Cell)**	**0.2**	**2**	**10**	**100**	**400**
1	0.094	0.387	0.400	0.433	0.468	0.395	0.352
2	0.092	0.366	0.417	0.447	0.432	0.376	0.337
3	0.100	0.392	0.468	0.389	0.493	0.402	0.343
Total	0.286	1.145	1.285	1.269	1.393	1.173	1.032
Mean	0.095	0.382	0.428	0.423	0.464	0.391	0.344
Cell Viability (%)			116.3	114.4	128.9	103.3	86.8

HYDROXYCHLOROQUINE-AZITHROMYCIN

HYDROXYCHLOROQUINE (μg/mL)							
**No**	**Control (Media)**	**Control (Cell)**	**0.2**	**2**	**10**	**100**	**400**
1	0.092	0.382	0.334	0.351	0.359	0.217	0.290
2	0.085	0.379	0.334	0.348	0.361	0.221	0.280
3	0.092	0.375	0.343	0.359	0.367	0.235	0.294
Total	0.269	1.136	1.011	1.058	1.087	0.673	0.864
Mean	0.090	0.379	0.337	0.353	0.362	0.224	0.288
Cell Viability (%)			85.6	91.0	94.4	46.6	68.6

AZITHROMYCIN (μg/mL)							
**No**	**Control (Media)**	**Control (Cell)**	**0.2**	**2**	**10**	**100**	**400**
1	0.092	0.382	0.345	0.363	0.393	0.406	0.285
2	0.085	0.379	0.345	0.364	0.389	0.417	0.291
3	0.092	0.375	0.355	0.359	0.390	0.397	0.290
Total	0.269	1.136	1.045	1.086	1.172	1.220	0.866
Mean	0.090	0.379	0.348	0.362	0.391	0.407	0.289
Cell Viability (%)			89.5	94.2	104.2	109.7	68.9

HCQ-AZI COMBINATIONS (1/1)							
**No**	**Control (Media)**	**Control (Cell)**	**0.2**	**2**	**10**	**100**	**400**
1	0.092	0.382	0.321	0.363	0.371	0.230	0.191
2	0.085	0.379	0.332	0.344	0.379	0.217	0.179
3	0.092	0.375	0.324	0.357	0.381	0.229	0.179
Total	0.269	1.136	0.977	1.064	1.131	0.676	0.549
Mean	0.090	0.379	0.326	0.355	0.377	0.225	0.183
Cell Viability (%)			81.7	91.7	99.4	47.0	32.3

HCQ-AZI COMBINATIONS (1/2)							
**No**	**Control (Media)**	**Control (Cell)**	**0.2**	**2**	**10**	**100**	**400**
1	0.092	0.382	0.361	0.389	0.401	0.247	0.310
2	0.085	0.379	0.354	0.369	0.391	0.262	0.273
3	0.092	0.375	0.360	0.375	0.391	0.244	0.273
Total	0.269	1.136	1.075	1.133	1.183	0.753	0.856
Mean	0.090	0.379	0.358	0.378	0.394	0.251	0.285
Cell Viability (%)			93.0	99.7	105.4	55.8	67.7

HCQ (µg/mL) + AZI 50 µg/mL							
**No**	**Control (Media)**	**Control (Cell)**	**0.2**	**2**	**10**	**100**	**400**
1	0.092	0.382	0.344	0.379	0.382	0.226	0.256
2	0.085	0.379	0.388	0.385	0.346	0.218	0.224
3	0.092	0.375	0.387	0.384	0.363	0.221	0.260
Total	0.269	1.136	1.119	1.148	1.091	0.665	0.740
Mean	0.090	0.379	0.373	0.383	0.364	0.222	0.247
Cell Viability (%)			98.0	101.4	94.8	45.7	54.3

HCQ 6 µg/mL + AZI (µg/mL)							
**No**	**Control (Media)**	**Control (Cell)**	**0.2**	**2**	**10**	**100**	**400**
1	0.092	0.382	0.422	0.420	0.425	0.407	0.201
2	0.085	0.379	0.430	0.448	0.425	0.357	0.255
3	0.092	0.375	0.424	0.411	0.417	0.366	0.239
Total	0.269	1.136	1.276	1.279	1.267	1.130	0.695
Mean	0.090	0.379	0.425	0.426	0.422	0.377	0.232
Cell Viability (%)			116.1	116.5	115.1	99.3	49.2

HYDROXYCHLOROQUINE-DOXYCYCLINE

HYDROXYCHLOROQUINE (µg/mL)							
**No**	**Control (Media)**	**Control (Cell)**	**0.2**	**2**	**10**	**100**	**400**
1	0.092	0.558	0.514	0.512	0.534	0.331	0.326
2	0.085	0.501	0.535	0.512	0.531	0.344	0.356
3	0.092	0.531	0.503	0.527	0.510	0.414	0.338
Total	0.269	1.590	1.552	1.551	1.575	1.089	1.020
Mean	0.090	0.530	0.517	0.517	0.525	0.363	0.340
Cell viability (%)			97.1	97.0	98.9	62.1	56.9

DOXYCYCLINE (µg/mL)							
**No**	**Control (Media)**	**Control (Cell)**	**0.2**	**2**	**10**	**100**	**400**
1	0.092	0.558	0.526	0.526	0.608	0.608	0.508
2	0.085	0.532	0.523	0.625	0.617	0.623	0.527
3	0.092	0.514	0.514	0.611	0.611	0.636	0.522
Total	0.269	1.604	1.563	1.762	1.836	1.867	1.557
Mean	0.090	0.535	0.521	0.587	0.612	0.622	0.519
Cell viability (%)			96.9	111.8	117.4	119.7	96.5

HCQ-DOXY COMBINATIONS (1/1)							
**No**	**Control (Media)**	**Control (Cell)**	**0.2**	**2**	**10**	**100**	**400**

1	0.092	0.558	0.548	0.534	0.512	0.426	0.364
2	0.085	0.532	0.510	0.579	0.507	0.410	0.335
3	0.092	0.514	0.469	0.519	0.513	0.398	0.389
Total	0.269	1.604	1.527	1.632	1.532	1.234	1.088
Mean	0.090	0.535	0.509	0.544	0.511	0.411	0.363
Cell viability (%)			94.2	102.1	94.6	72.3	61.4

HCQ-DOXY COMBINATIONS (1/2)							
**No**	**Control (Media)**	**Control (Cell)**	**0.2**	**2**	**10**	**100**	**400**
1	0.092	0.558	0.506	0.574	0.524	0.519	0.347
2	0.085	0.532	0.491	0.588	0.549	0.486	0.346
3	0.092	0.514	0.546	0.593	0.556	0.528	0.341
Total	0.269	1.604	1.543	1.755	1.629	1.533	1.034
Mean	0.090	0.535	0.514	0.585	0.543	0.511	0.345
Cell viability (%)			95.4	111.3	101.9	94.7	57.3

HCQ (μg/mL) + DOXY 2 μg/mL							
**No**	**Control (Media)**	**Control (Cell)**	**0.2**	**2**	**10**	**100**	**400**
1	0.092	0.558	0.524	0.543	0.532	0.424	0.430
2	0.085	0.532	0.270	0.523	0.516	0.508	0.425
3	0.092	0.514	0.513	0.528	0.523	0.423	0.443
Total	0.269	1.604	1.307	1.594	1.571	1.355	1.298
Mean	0.090	0.535	0.436	0.531	0.524	0.452	0.433
Cell viability (%)			77.8	99.3	97.5	81.4	77.1

HCQ 6 μg/mL + DOXY (μg/mL)							
**No**	**Control (Media)**	**Control (Cell)**	**0.2**	**2**	**10**	**100**	**400**
1	0.092	0.558	0.555	0.619	0.619	0.595	0.521
2	0.085	0.532	0.582	0.624	0.622	0.542	0.527
3	0.092	0.514	0.588	0.623	0.581	0.593	0.532
Total	0.269	1.604	1.725	1.866	1.822	1.730	1.580
Mean	0.090	0.535	0.575	0.622	0.607	0.577	0.527
Cell viability (%)			109.1	119.6	116.3	109.4	98.2

FAVIPIRAVIR-AZITHROMYCIN

FAVIPIRAVIR (μg/mL)							
**No**	**Control (Media)**	**Control (Cell)**	**0.2**	**2**	**10**	**100**	**400**
1	0.094	0.354	0.375	0.367	0.375	0.376	0.410
2	0.092	0.363	0.367	0.372	0.380	0.397	0.432
3	0.100	0.357	0.371	0.364	0.388	0.379	0.438
Total	0.286	1.074	1.113	1.103	1.143	1.152	1.280
Mean	0.095	0.358	0.371	0.368	0.381	0.384	0.427
Cell Viability (%)			104.9	103.7	108.8	109.9	126.1

AZITHROMYCIN (μg/mL)							
**No**	**Control (Media)**	**Control (Cell)**	**0.2**	**2**	**10**	**100**	**400**
1	0.094	0.316	0.358	0.344	0.355	0.409	0.293
2	0.092	0.344	0.383	0.373	0.363	0.442	0.321
3	0.100	0.349	0.377	0.345	0.367	0.438	0.329

Total	0.286	1.009	1.118	1.062	1.085	1.289	0.943
Mean	0.095	0.336	0.373	0.354	0.362	0.430	0.314
Cell Viability (%)			115.1	107.3	110.5	138.7	90.9

FAVI-AZI COMBINATIONS (2/1)							
**No**	**Control (Media)**	**Control (Cell)**	**0.2**	**2**	**10**	**100**	**400**
1	0.094	0.316	0.378	0.385	0.415	0.396	0.355
2	0.092	0.344	0.364	0.390	0.424	0.380	0.354
3	0.100	0.349	0.377	0.407	0.425	0.403	0.342
Total	0.286	1.009	1.119	1.182	1.264	1.179	1.051
Mean	0.095	0.336	0.373	0.394	0.421	0.393	0.350
Cell Viability (%)			115.2	123.9	135.3	123.5	105.8

FAVI-AZI COMBINATIONS (1/3)							
**No**	**Control (Media)**	**Control (Cell)**	**0.2**	**2**	**10**	**100**	**400**
1	0.094	0.316	0.402	0.471	0.510	0.383	0.362
2	0.092	0.344	0.402	0.458	0.500	0.401	0.365
3	0.100	0.349	0.404	0.491	0.512	0.389	0.396
Total	0.286	1.009	1.208	1.420	1.522	1.173	1.123
Mean	0.095	0.336	0.403	0.473	0.507	0.391	0.374
Cell Viability (%)			127.5	156.8	171.0	122.7	115.8

FAVI (µg/mL) + AZI 50 µg/mL							
**No**	**Control (Media)**	**Control (Cell)**	**0.2**	**2**	**10**	**100**	**400**
1	0.094	0.316	0.397	0.380	0.404	0.383	0.531
2	0.092	0.344	0.409	0.375	0.414	0.387	0.563
3	0.100	0.349	0.398	0.374	0.417	0.392	0.459
Total	0.286	1.009	1.204	1.129	1.235	1.162	1.553
Mean	0.095	0.336	0.401	0.376	0.412	0.387	0.518
Cell Viability (%)			127.0	116.6	131.3	121.2	175.2

FAVI 66 µg/mL + AZI (µg/mL)							
**No**	**Control (Media)**	**Control (Cell)**	**0.2**	**2**	**10**	**100**	**400**
1	0.094	0.316	0.360	0.384	0.413	0.419	0.390
2	0.092	0.344	0.406	0.397	0.395	0.435	0.349
3	0.100	0.349	0.402	0.385	0.442	0.409	0.321
Total	0.286	1.009	1.168	1.166	1.250	1.263	1.060
Mean	0.095	0.336	0.389	0.389	0.417	0.421	0.353
Cell Viability (%)			122.0	121.7	133.3	135.1	107.1

HYDROXYCHLOROQUINE-FAVIPIRAVIR

HYDROXYCHLOROQUINE (µg/mL)							
**No**	**Control (Media)**	**Control (Cell)**	**0.2**	**2**	**10**	**100**	**400**
1	0.092	0.470	0.265	0.306	0.403	0.104	0.119
2	0.085	0.424	0.275	0.454	0.429	0.157	0.126
3	0.092	0.455	0.285	0.393	0.449	0.151	0.126
Total	0.269	1.349	0.825	1.153	1.281	0.412	0.371
Mean	0.090	0.450	0.275	0.384	0.427	0.137	0.124
Cell Viability (%)			51.5	81.9	93.7	13.3	9.5

FAVIPIRAVIR (µg/mL)							
**No**	**Control (Media)**	**Control (Cell)**	**0.2**	**2**	**10**	**100**	**400**
1	0.092	0.470	0.476	0.362	0.337	0.305	0.317
2	0.085	0.424	0.447	0.336	0.387	0.307	0.345
3	0.092	0.455	0.345	0.347	0.327	0.372	0.328
Total	0.269	1.349	1.268	1.045	1.051	0.984	0.990
Mean	0.090	0.450	0.423	0.348	0.350	0.328	0.330
Cell Viability (%)			92.5	71.9	72.4	66.2	66.8

HCQ-FAVI COMBINATIONS (1/5)							
**No**	**Control (Media)**	**Control (Cell)**	**0.2**	**2**	**10**	**100**	**400**
1	0.092	0.470	0.432	0.388	0.455	0.168	0.184
2	0.085	0.424	0.407	0.371	0.395	0.183	0.164
3	0.092	0.455	0.393	0.412	0.428	0.184	0.174
Total	0.269	1.349	1.232	1.171	1.278	0.535	0.522
Mean	0.090	0.450	0.411	0.390	0.426	0.178	0.174
Cell Viability (%)			89.2	83.5	93.4	24.7	23.4

HCQ-FAVI COMBINATIONS (1/10)							
**No**	**Control (Media)**	**Control (Cell)**	**0.2**	**2**	**10**	**100**	**400**
1	0.092	0.470	0.472	0.372	0.398	0.219	0.178
2	0.085	0.424	0.401	0.373	0.395	0.198	0.279
3	0.092	0.455	0.481	0.385	0.401	0.287	0.227
Total	0.269	1.349	1.354	1.130	1.194	0.704	0.684
Mean	0.090	0.450	0.451	0.377	0.398	0.235	0.228
Cell Viability (%)			100.5	79.7	85.7	40.3	38.4

HCQ 6 µg/mL+ FAVI (µg/mL)							
**No**	**Control (Media)**	**Control (Cell)**	**0.2**	**2**	**10**	**100**	**400**
1	0.092	0.470	0.359	0.354	0.425	0.374	0.415
2	0.085	0.424	0.441	0.356	0.451	0.367	0.405
3	0.092	0.455	0.439	0.416	0.489	0.362	0.397
Total	0.269	1.349	1.239	1.126	1.365	1.103	1.217
Mean	0.090	0.450	0.413	0.375	0.455	0.368	0.406
Cell Viability (%)			89.8	79.4	101.5	77.2	87.8

HCQ (µg/mL) + FAVI 66 µg/mL							
**No**	**Control (Media)**	**Control (Cell)**	**0.2**	**2**	**10**	**100**	**400**
1	0.092	0.470	0.331	0.304	0.342	0.152	0.130
2	0.085	0.424	0.364	0.357	0.329	0.142	0.132
3	0.092	0.455	0.333	0.322	0.417	0.148	0.135
Total	0.269	1.349	1.028	0.983	1.088	0.442	0.397
Mean	0.090	0.450	0.343	0.328	0.363	0.147	0.132
Cell Viability (%)			70.3	66.1	75.8	16.0	11.9

HYDROXYCHLOROQUINE-LOPIRITO

HYDROXYCHLOROQUINE (µg/mL)							
**No**	**Control (Media)**	**Control (Cell)**	**0.2**	**2**	**10**	**100**	**400**

1	0.092	0.436	0.211	0.294	0.378	0.205	0.117
2	0.085	0.424	0.214	0.330	0.336	0.211	0.105
3	0.092	0.445	0.216	0.284	0.398	0.195	0.123
Total	0.269	1.305	0.641	0.908	1.112	0.611	0.345
Mean	0.090	0.435	0.214	0.303	0.371	0.204	0.115
Cell Viability (%)			35.9	61.7	81.4	33.0	7.4

LOPIRITO (as LOPINAVIR in µg/mL)							
**No**	**Control (Media)**	**Control (Cell)**	**0.2**	**2**	**10**	**100**	**400**
1	0.092	0.436	0.314	0.299	0.275	0.239	0.237
2	0.085	0.424	0.345	0.267	0.279	0.238	0.266
3	0.092	0.445	0.395	0.294	0.294	0.260	0.253
Total	0.269	1.305	1.054	0.860	0.848	0.737	0.756
Mean	0.090	0.435	0.351	0.287	0.283	0.246	0.252
Cell Viability (%)			75.8	57.1	55.9	45.2	47.0

HCQ-LOPIRITO COMBINATIONS (1/1)							
**No**	**Control (Media)**	**Control (Cell)**	**0.2**	**2**	**10**	**100**	**400**
1	0.092	0.470	0.333	0.342	0.317	0.226	0.124
2	0.085	0.424	0.288	0.309	0.327	0.188	0.120
3	0.092	0.455	0.297	0.323	0.310	0.187	0.128
Total	0.269	1.349	0.918	0.974	0.954	0.601	0.372
Mean	0.090	0.450	0.306	0.325	0.318	0.200	0.124
Cell Viability (%)			60.1	65.3	63.4	30.8	9.6

HCQ-LOPIRITO COMBINATIONS (1/2)							
**No**	**Control (Media)**	**Control (Cell)**	**0.2**	**2**	**10**	**100**	**400**
1	0.092	0.436	0.217	0.309	0.253	0.215	0.152
2	0.085	0.424	0.274	0.289	0.278	0.240	0.143
3	0.092	0.445	0.303	0.296	0.290	0.244	0.150
Total	0.269	1.305	0.794	0.894	0.821	0.699	0.445
Mean	0.090	0.435	0.265	0.298	0.274	0.233	0.148
Cell Viability (%)			50.7	60.3	53.3	41.5	17.0

HCQ (µg/mL) + LOPIRITO 8 µg/mL							
**No**	**Control (Media)**	**Control (Cell)**	**0.2**	**2**	**10**	**100**	**400**
1	0.092	0.436	0.319	0.321	0.319	0.241	0.112
2	0.085	0.424	0.297	0.325	0.341	0.197	0.113
3	0.092	0.445	0.340	0.298	0.319	0.198	0.113
Total	0.269	1.305	0.956	0.944	0.979	0.636	0.338
Mean	0.090	0.435	0.319	0.315	0.326	0.212	0.113
Cell Viability (%)			66.3	65.2	68.5	35.4	6.7

HCQ 6 µg/mL + LOPIRITO (µg/mL)							
**No**	**Control (Media)**	**Control (Cell)**	**0.2**	**2**	**10**	**100**	**400**
1	0.092	0.436	0.398	0.332	0.285	0.337	0.312
2	0.085	0.424	0.374	0.333	0.316	0.310	0.316
3	0.092	0.445	0.377	0.347	0.308	0.303	0.283
Total	0.269	1.305	1.149	1.012	0.909	0.950	0.628
Mean	0.090	0.435	0.383	0.337	0.303	0.317	0.209
Cell Viability (%)			84.9	71.7	61.8	65.7	34.7
