# Supplementary material for: An in vitro study of dual drug combinations of anti-viral agents, antibiotics, and/or hydroxychloroquine against the SARS-CoV-2 virus isolated from hospitalized patients in Surabaya, Indonesia
Source: PLoS One. 2021 Jun 18;16(6):e0252302. doi: 10.1371/journal.pone.0252302 (PMC8213153; doi:10.1371/journal.pone.0252302)
Supplement: S2 Table — (PDF) [file pone.0252302.s002.pdf]

S2 Table. The average virus titer of of Vero cells infected with SARS-CoV-2 isolates an multiplicity of infection (MoI) value of 0.04 at 24, 48, and 72 hours incubated with single and drug combinations (n=2).

**DRUGS**		**DRUG CONCENTRATION 1 µg/mL**			**DRUG CONCENTRATION 15 µg/mL**			**DRUG CONCENTRATION 37.5 µg/mL**		
		**24-h**	**48-h**	**72-h**	**24-h**	**48-h**	**72-h**	**24-h**	**48-h**	**72-h**
Lopinavir/Ritonavir	Virus Titer (viral protein fg/mL)	36.65	ND	ND	38.82	ND	38.09	ND	ND	ND
**DRUGS**		**DRUG CONCENTRATION 15 µg/mL**			**DRUG CONCENTRATION 62.5 µg/mL**			**DRUG CONCENTRATION 125 µg/mL**		
		**24-h**	**48-h**	**72-h**	**24-h**	**48-h**	**72-h**	**24-h**	**48-h**	**72-h**
Azithromycin	Virus Titer (viral protein fg/mL)	38.18	ND	ND	ND	38.78	ND	39.15	ND	ND
**DRUGS**		**DRUG CONCENTRATION 0.5 µg/mL**			**DRUG CONCENTRATION 4 µg/mL**			**DRUG CONCENTRATION 8 µg/mL**		
		**24-h**	**48-h**	**72-h**	**24-h**	**48-h**	**72-h**	**24-h**	**48-h**	**72-h**
Clarithromycin	Virus Titer (viral protein fg/mL)	38.47	ND	ND	ND	ND	ND	38.03	39.09	37.46
**DRUGS**		**DRUG CONCENTRATION 1 µg/mL**			**DRUG CONCENTRATION 15 µg/mL**			**DRUG CONCENTRATION 37.5 µg/mL**		
		**24-h**	**48-h**	**72-h**	**24-h**	**48-h**	**72-h**	**24-h**	**48-h**	**72-h**
Doxycycline	Virus Titer (viral protein fg/mL)	38.42	ND	ND	36.90	37.85	39.15	ND	ND	ND
**DRUGS**		**DRUG CONCENTRATION 1 µg/mL**			**DRUG CONCENTRATION 15 µg/mL**			**DRUG CONCENTRATION 37.5 µg/mL**		
		**24-h**	**48-h**	**72-h**	**24-h**	**48-h**	**72-h**	**24-h**	**48-h**	**72-h**
Hydroxychloroquine	Virus Titer (viral protein fg/mL)	38.64	ND	37.69	ND	37.86	ND	39.04	ND	ND
**DRUGS**		**DRUG CONCENTRATION 10 µg/mL**			**DRUG CONCENTRATION 15 µg/mL**			**DRUG CONCENTRATION 37.5 µg/mL**		
		**24-h**	**48-h**	**72-h**	**24-h**	**48-h**	**72-h**	**24-h**	**48-h**	**72-h**
Favipiravir	Virus Titer (viral protein fg/mL)	ND	37.37	ND	38.87	ND	ND	ND	38.84	ND
**DRUGS**		**DRUG CONCENTRATION 25 µg/mL**			**DRUG CONCENTRATION 50 µg/mL**			**DRUG CONCENTRATION 100 µg/mL**		
		**24-h**	**48-h**	**72-h**	**24-h**	**48-h**	**72-h**	**24-h**	**48-h**	**72-h**
Lopinavir/Ritonavir + Azithromycin (1:2)	Virus Titer (viral protein fg/mL)	ND	40.34	ND	ND	ND	ND	ND	36.93	ND
**DRUGS**		**DRUG CONCENTRATION 1 µg/mL**			**DRUG CONCENTRATION 10 µg/mL**			**DRUG CONCENTRATION 30 µg/mL**		
		**24-h**	**48-h**	**72-h**	**24-h**	**48-h**	**72-h**	**24-h**	**48-h**	**72-h**
Lopinavir/Ritonavir + Clarithromycin (1:1)	Virus Titer (viral protein fg/mL)	ND	38.26	ND	35.76	ND	ND	39.42	ND	ND

DRUGS		DRUG CONCENTRATION 5 µg/mL			DRUG CONCENTRATION 10 µg/mL			DRUG CONCENTRATION 15 µg/mL		
		**24-h**	**48-h**	**72-h**	**24-h**	**48-h**	**72-h**	**24-h**	**48-h**	**72-h**
Lopinavir/Ritonavir + Doxycycline (1:1)	Virus Titer (viral protein fg/mL)	38.61	41.86	ND	38.55	ND	ND	ND	39.06	ND
**DRUGS**		**DRUG CONCENTRATION 25 µg/mL**			**DRUG CONCENTRATION 50 µg/mL**			**DRUG CONCENTRATION 100 µg/mL**		
		**24-h**	**48-h**	**72-h**	**24-h**	**48-h**	**72-h**	**24-h**	**48-h**	**72-h**
Hydroxychloroquine + Azithromycin (1:2)	Virus Titer (viral protein fg/mL)	37.26	ND		ND	38.41		ND	ND	
**DRUGS**		**DRUG CONCENTRATION 10 µg/mL**			**DRUG CONCENTRATION 25 µg/mL**			**DRUG CONCENTRATION 50 µg/mL**		
		**24-h**	**48-h**	**72-h**	**24-h**	**48-h**	**72-h**	**24-h**	**48-h**	**72-h**
Hydroxychloroquine + Doxycycline (1:2)	Virus Titer (viral protein fg/mL)	37.94	ND	36.20	38.38	ND	ND	ND	ND	36.87
**DRUGS**		**DRUG CONCENTRATION 25 µg/mL**			**DRUG CONCENTRATION 50 µg/mL**			**DRUG CONCENTRATION 200 µg/mL**		
		**24-h**	**48-h**	**72-h**	**24-h**	**48-h**	**72-h**	**24-h**	**48-h**	**72-h**
Favipiravir + Azithromycin (2:1)	Virus Titer (viral protein fg/mL)	38.98	ND	35.79	ND	39.18	36.97	39.00	ND	ND
**DRUGS**		**DRUG CONCENTRATION 35 µg/mL**			**DRUG CONCENTRATION 75 µg/mL**			**DRUG CONCENTRATION 150 µg/mL**		
		**24-h**	**48-h**	**72-h**	**24-h**	**48-h**	**72-h**	**24-h**	**48-h**	**72-h**
Hydroxychloroquine + Favipiravir (1:10)	Virus Titer (viral protein fg/mL)	37.62	37.74	ND	ND	39.13	ND	ND	ND	ND
**DRUGS**		**DRUG CONCENTRATION 10 µg/mL**			**DRUG CONCENTRATION 25 µg/mL**			**DRUG CONCENTRATION 50 µg/mL**		
		**24-h**	**48-h**	**72-h**	**24-h**	**48-h**	**72-h**	**24-h**	**48-h**	**72-h**
Hydroxychloroquine + Lopinavir/Ritonavir (1:2)	Virus Titer (viral protein fg/mL)	37.95	39.26	ND	38.38	36.96	37.38	ND	ND	37.41

NOTE:

ND means not detected
