# Supplementary material for: An in vitro study of dual drug combinations of anti-viral agents, antibiotics, and/or hydroxychloroquine against the SARS-CoV-2 virus isolated from hospitalized patients in Surabaya, Indonesia
Source: PLoS One. 2021 Jun 18;16(6):e0252302. doi: 10.1371/journal.pone.0252302 (PMC8213153; doi:10.1371/journal.pone.0252302)
Supplement: S3 Table — (PDF) [file pone.0252302.s003.pdf]

S3 Table. The cytokine levels of Vero cells infected with SARS-CoV-2 isolates an multiplicity of infection (MoI) value of 0.04 at 24, 48, and 72 hours incubated with single and drug combinations (n=2).

DRUGS	CYTOKINES	DRUG CONCENTRATION 1 μg/mL			DRUG CONCENTRATION 15 μg/mL			DRUG CONCENTRATION 37.5 μg/mL		
		24-h	48-h	72-h	24-h	48-h	72-h	24-h	48-h	72-h
Lopinavir/Ritonavir	IL-10 (pg/mL)	32.43 ± 4.09	87.02 ± 2.83	465.82 ± 1.39	28.31 ± 1.64	80.66 ± 1.53	473.94 ± 2.09	24.75 ± 1.56	80.14 ± 0.10	478.03 ± 1.43
	IL-6 (ng/L)	16.68 ± 1.22	23.63 ± 1.63	30.49 ± 0.45	18.08 ± 2.87	28.56 ± 1.08	39.44 ± 0.66	18.70 ± 1.48	45.31 ± 2.05	53.56 ± 0.43
	TNF-α (pg/mL)	0.88 ± 0.02	336.12 ± 1.35	381.85 ± 1.13	38.75 ± 1.18	488.38 ± 1.28	571.19 ± 0.00	49.56 ± 0.97	372.20 ± 1.73	444 ± 3.88
**DRUGS**	**CYTOKINES**	**DRUG CONCENTRATION 15 μg/mL**			**DRUG CONCENTRATION 62.5 μg/mL**			**DRUG CONCENTRATION 125 μg/mL**		
		**24-h**	**48-h**	**72-h**	**24-h**	**48-h**	**72-h**	**24-h**	**48-h**	**72-h**
Azithromycin	IL-10 (pg/mL)	32.78 ± 1.18	152.34 ± 1.72	609.92 ± 1.48	53.50 ± 1.85	91.92 ± 1.45	541.70 ± 11.47	28.64 ± 0.95	77.04 ± 2.77	400.04 ± 28.27
	IL-6 (ng/L)	14.55 ± 2.22	33.9 8± 0.64	565.92 ± 0.00	15.92 ± 0.57	33.47 ± 3.98	529.11 ± 0.95	16.68 ± 0.46	30.24 ± 2.61	396.84 ± 3.92
	TNF-α (pg/mL)	6.994 ± 1.32	478 ± 1.50	777.34 ± 1.67	86.00 ± 2.84	381.30 ± 1.28	641.33 ± 0.00	145.12 ± 1.39	319.99 ± 1.14	447.94 ± 0.99
**DRUGS**	**CYTOKINES**	**DRUG CONCENTRATION 0.5 μg/mL**			**DRUG CONCENTRATION 4 μg/mL**			**DRUG CONCENTRATION 8 μg/mL**		
		**24-h**	**48-h**	**72-h**	**24-h**	**48-h**	**72-h**	**24-h**	**48-h**	**72-h**
Clarithromycin	IL-10 (pg/mL)	36.80 ± 1.13	105.49 ± 1.32	507.18 ± 0.00	36.80 ± 0.08	58.15 ± 1.41	498.75 ± 0.00	24.75 ± 0.58	79.10 ± 0.00	445.89 ± 0.07
	IL-6 (ng/L)	15.61 ± 0.85	28.56 ± 0.62	48.22 ± 0.35	14.85 ± 0.51	30.07 ± 0.09	51.13 ± 0.94	15.61 ± 0.57	34.67 ± 0.50	64.93 ± 2.50
	TNF-α (pg/mL)	20.39 ± 0.57	426.37 ± 1.40	612.06 ± 1.54	100.04 ± 1.44	404.07 ± 0.10	609.56 ± 0.14	43.92 ± 1.13	382.62 ± 0.86	609.56 ± 0.14
**DRUGS**	**CYTOKINES**	**DRUG CONCENTRATION 1 μg/mL**			**DRUG CONCENTRATION 15 μg/mL**			**DRUG CONCENTRATION 37.5 μg/mL**		
		**24-h**	**48-h**	**72-h**	**24-h**	**48-h**	**72-h**	**24-h**	**48-h**	**72-h**
Doxycycline	IL-10 (pg/mL)	35.31 ± 0.22	219.74 ± 0.08	415.82 ± 0.23	35.31 ± 1.53	219.74 ± 1.41	702.97 ± 0.07	28.98 ± 1.40	79.10 ± 1.41	438.06 ± 0.00
	IL-6 (ng/L)	21.06 ± 1.43	36.93 ± 0.93	40.56 ± 0.56	13.34 ± 0.86	37.98 ± 0.94	43.01 ± 0.05	17.45 ± 0.12	40.48 ± 0.14	50.92 ± 0.18
	TNF-α (pg/mL)	1.98 ± 0.00	408.19 ± 0.77	511.16 ± 3.09	1.98 ± 0.84	408.19 ± 0.84	529.12 ± 0.46	119.61 ± 8.82	420.72 ± 0.77	556.55 ± 2.01
**DRUGS**	**CYTOKINES**	**DRUG CONCENTRATION 1 μg/mL**			**DRUG CONCENTRATION 15 μg/mL**			**DRUG CONCENTRATION 37.5 μg/mL**		
		**24-h**	**48-h**	**72-h**	**24-h**	**48-h**	**72-h**	**24-h**	**48-h**	**72-h**
Hydroxychloroquine	IL-10 (pg/mL)	15.36 ± 0.75	96.36 ± 0.71	329.79 ± 0.08	32.43 ± 1.19	177.64 ± 11.46	258.02 ± 9.73	29.65 ± 0.66	91.37 ± 1.41	129.95 ± 0.04
	IL-6 (ng/L)	15.91 ± 0.99	36.41 ± 2.01	47.64 ± 2.15	15.91 ± 1.41	36.41 ± 0.89	39.80 ± 0.48	19.48 ± 1.39	34.33 ± 1.47	40.85 ± 1.58
	TNF-α (pg/mL)	4.21 ± 0.31	446.60 ± 13.56	548.17 ± 1.44	167.81 ± 9.91	458.44 ± 82.79	411.10 ± 0.18	90.50 ± 3.03	448.06 ± 15.27	560.77 ± 4.83
**DRUGS**	**CYTOKINES**	**DRUG CONCENTRATION 10 μg/mL**			**DRUG CONCENTRATION 15 μg/mL**			**DRUG CONCENTRATION 37.5 μg/mL**		
		**24-h**	**48-h**	**72-h**	**24-h**	**48-h**	**72-h**	**24-h**	**48-h**	**72-h**
Favipiravir	IL-10 (pg/mL)	31.38 ± 1.80	125.32 ± 1.57	465.82 ± 0.57	27.97 ±0.29	100.89 ± 1.28	411.24 ± 0.24	23.20 ± 2.55	93.58 ± 0.32	411.24 ± 1.34
	IL-6 (ng/L)	14.09 ± 1.53	37.28 ± 0.37	60.21 ± 0.49	22.18 ±1.09	39.75 ± 1.67	68.61 ± 1.04	16.84 ± 0.28	37.81 ± 2.41	64.99 ± 0.15

	TNF-α (pg/mL)	3.09 ± 1.23	416.51 ±1.28	711.37 ± 0.70	98.18 ±1.55	467.47 ± 3.11	725.48 ± 3.51	86.00 ± 1.26	517.79 ± 1.27	802.57 ± 2.98
**DRUGS**	**CYTOKINES**	**DRUG CONCENTRATION 25 µg/mL**			**DRUG CONCENTRATION 50 µg/mL**			**DRUG CONCENTRATION 100 µg/mL**		
		**24-h**	**48-h**	**72-h**	**24-h**	**48-h**	**72-h**	**24-h**	**48-h**	**72-h**
Lopinavir/Ritonavir + Azithromycin (1:2)	IL-10 (pg/mL)	25.06 ± 1.06	99.75 ± 0.31	238.00 ± 0.00	34.95 ±0.58	122.24 ± 1.42	230.00 ± 1.41	20.81 ± 1.93	81.71 ± 0.49	97.83 ± 0.04
	IL-6 (ng/L)	15.92 ± 0.58	36.23 ±0.66	50.01 ± 0.97	16.07 ±1.84	39.92 ± 0.28	75.57 ± 0.85	15.76 ± 1.08	37.63 ± 1.36	63.29 ± 0.61
	TNF-α (pg/mL)	31.80 ± 1.27	389.23 ±1.40	414.58 ± 2.27	95.18 ±1.16	462.94 ± 1.88	567.82 ± 2.31	133.74 ± 0.77	377.38 ± 1.41	419.63 ± 0.79
**DRUGS**	**CYTOKINES**	**DRUG CONCENTRATION 1 µg/mL**			**DRUG CONCENTRATION 10 µg/mL**			**DRUG CONCENTRATION 30 µg/mL**		
		**24-h**	**48-h**	**72-h**	**24-h**	**48-h**	**72-h**	**24-h**	**48-h**	**72-h**
Lopinavir/Ritonavir + Clarithromycin (1:1)	IL-10 (pg/mL)	25.06 ± 1.06	99.75 ± 0.31	238.00 ± 0.00	34.95 ±0.58	122.24 ± 1.42	230.00 ± 1.41	20.81 ±1.93	81.71 ± 0.49	97.83 ± 0.04
	IL-6 (ng/L)	15.92 ± 0.58	36.23 ± 0.66	50.01 ± 0.97	16.07 ±1.84	39.92 ± 0.28	75.57 ± 0.85	15.76 ±1.08	37.63 ± 1.36	63.29 ± 0.61
	TNF-α (pg/mL)	31.80 ± 1.27	389.23 ±1.40	414.58 ± 2.27	95.18 ±1.16	462.94 ± 1.88	567.82 ± 2.31	133.74 ± 0.77	377.38 ± 1.41	419.63 ± 0.79
**DRUGS**	**CYTOKINES**	**DRUG CONCENTRATION 5 µg/mL**			**DRUG CONCENTRATION 10 µg/mL**			**DRUG CONCENTRATION 15 µg/mL**		
		**24-h**	**48-h**	**72-h**	**24-h**	**48-h**	**72-h**	**24-h**	**48-h**	**72-h**
Lopinavir/Ritonavir + Doxycycline (1:1)	IL-10 (pg/mL)	36.05 ± 0.35	151.01 ±0.2	457.73 ± 0.21	30.68 ± 100.32	100.32 ± 0.64	528.60 ± 0.00	33.14 ± 1.41	94.7 ± 0 .44	449.80 ± 0.00
	IL-6 (ng/L)	20.27 ± 0.88	32.27 ± 1.88	47.43 ± 0.90	15.76 ±0.41	47.32 ± 1.13	70.16 ± 2.26	14.84 ± 1.58	49.71 ± 1.27	73.56 ± 1.13
	TNF-α (pg/mL)	69.64 ± 0.86	378.68 ±0.45	398.95 ± 1.15	29.68 ±0.57	426.37 ± 3.82	576.78 ± 3.28	52.56 ± 0.91	419.31 ± 2.40	464.37 ± 3.98
**DRUGS**	**CYTOKINES**	**DRUG CONCENTRATION 25 µg/mL**			**DRUG CONCENTRATION 50 µg/mL**			**DRUG CONCENTRATION 100 µg/mL**		
		**24-h**	**48-h**	**72-h**	**24-h**	**48-h**	**72-h**	**24-h**	**48-h**	**72-h**
Hydroxychloroquine + Azithromycin (1:2)	IL-10 (pg/mL)	27.64 ± 0.74	181.86 ±0.97	465.82 ± 22.35	28.64 ±1.98	119.18 ± 1.52	595.90 ± 14.28	23.20 ± 1.44	92.47 ± 1.42	407.49 ± 1.41
	IL-6 (ng/L)	14.70 ± 0.21	30.57 ± 0.08	61.56 ± 0.48	17.15 ±1.45	25.75 ± 1.45	57.53 ± 1.43	16.99 ± 1.56	37.81 ± 1.53	51.31 ± 1.68
	TNF-α (pg/mL)	52.56 ± 1.75	484.36 ± 0.61	813.06 ± 3.87	256.45 ±1.73	434.96 ± 1.57	795.85 ± 0.25	100.04 ± 1.43	410.95 ± 2.73	721.20 ± 1.14
**DRUGS**	**CYTOKINES**	**DRUG CONCENTRATION 10 µg/mL**			**DRUG CONCENTRATION 25 µg/mL**			**DRUG CONCENTRATION 50 µg/mL**		
		**24-h**	**48-h**	**72-h**	**24-h**	**48-h**	**72-h**	**24-h**	**48-h**	**72-h**
Hydroxychloroquine + Doxycycline (1:2)	IL-10 (pg/mL)	34.95 ± 0.01	107.25 ± 1.41	528.60 ± 1.34	44.61 ± 0.05	114.95 ± 1.34	438.05 ± 0.01	34.95 ± 1.34	143.12 ± 0.01	441.94 ± 0.04
	IL-6 (ng/L)	16.38 ± 0.73	131.08 ± 0.07	39.14 ± 0.01	17.92 ±1.32	128.15 ± 0.07	79.82 ± 0.08	17.45 ± 0.65	132.81 ± 0.14	45.04 ± 0.00
	TNF-α (pg/mL)	22.09 ± 0.14	423.54 ± 0.02	805.17 ± 2.10	31.80 ±0.03	377.38 ± 0.55	714.97 ± 0.01	49.56 ± 0.65	387.90 ± 0.07	586.07 ± 4.17
**DRUGS**	**CYTOKINES**	**DRUG CONCENTRATION 25 µg/mL**			**DRUG CONCENTRATION 50 µg/mL**			**DRUG CONCENTRATION 200 µg/mL**		
		**24-h**	**48-h**	**72-h**	**24-h**	**48-h**	**72-h**	**24-h**	**48-h**	**72-h**
Favipiravir + Azithromycin (2:1)	IL-10 (pg/mL)	35.68 ± 0.63	90.82 ±1.34	473.94 ± 15.61	41.02 ±1.41	177.63 ± 1.22	486.25 ± 14.33	35.31 ± 1.46	179.74 ±1.39	465.82 ± 1.48
	IL-6 (ng/L)	15.60 ± 0.89	101.84 ± 1.34	60.21 ± 1.55	44.76 ±1.26	111.51 ± 1.28	67 ± 1.41	20.27 ± 1.27	137.03 ± 2.24	60.19 ± 1.42
	TNF-α (pg/mL)	25.72 ± 0.69	373.48 ± 0.57	555.7 ± 5.74	41.27 ± 1.00	328.97 ± 1.41	518.90 ± 0.66	41.28 ± 1.55	399.98 ± 2.53	665.27 ± 2.51
**DRUGS**	**CYTOKINES**	**DRUG CONCENTRATION 35 µg/mL**			**DRUG CONCENTRATION 75 µg/mL**			**DRUG CONCENTRATION 150 µg/mL**		
		**24-h**	**48-h**	**72-h**	**24-h**	**48-h**	**72-h**	**24-h**	**48-h**	**72-h**

Hydroxychloroquine + Favipiravir (1:10)	IL-10 (pg/mL)	34.22 ± 0.01	96.36 ± 1.11	326.48 ± 1.77	37.05 ± 2.83	106.66 ± 0.03	614.63 ± 0.52	35.68 ± 0.01	32.22 ± 0.00	411.24 ± 0.00
	IL-6 (ng/L)	19.17 ± 0.03	40.81 ± 0.01	53.18 ± 2.86	19.96 ± 0.00	35.36 ± 0.31	56.18 ± 0.03	17.76 ± 0.00	53.07 ± 0.49	23.39 ± 2.14
	TNF-α (pg/mL)	52.56 ± 1.53	442.20 ± 0.01	698.97 ± 1.05	52.56 ± 0.04	394.58 ± 0.20	528.27± 0.00	43.92 ± 1.44	262.43 ± 0.00	326.45 ± 1.41
**DRUGS**	**CYTOKINES**	**DRUG CONCENTRATION 10 µg/mL**			**DRUG CONCENTRATION 25 µg/mL**			**DRUG CONCENTRATION 50 µg/mL**		
		**24-h**	**48-h**	**72-h**	**24-h**	**48-h**	**72-h**	**24-h**	**48-h**	**72-h**
Hydroxychloroquine + Lopinavir/Ritonavir (1:2)	IL-10 (pg/mL)	39.07 ± 0.14	89.19 ± 0.00	619.36 ± 1.75	29.99 ± 0.00	122.85 ± 0.16	392.67 ± 0.00	31.73 ± 1.04	14.44 ± 0.50	356.98 ± 7.07
	IL-6 (ng/L)	17.92 ± 0.04	31.42 ± 0.35	46.88 ± 0.17	17.76 ± 0.01	47.13 ± 0.22	59.04 ± 0.02	16.07 ± 1.83	37.11 ± 2.88	23.23 ± 0.80
	TNF-α (pg/mL)	34.02 ± 0.09	381.28 ± 0.03	819.89 ± 1.10	62.38 ± 0.07	404.07 ± 1.95	866.21	95.19 ± 6.77	368.34 ± 4.98	779.76 ± 5.86
